# Influence of Serotonin 5-HT_4_ Receptors on Responses to Cardiac Stressors in Transgenic Mouse Models

**DOI:** 10.3390/biomedicines9050569

**Published:** 2021-05-18

**Authors:** Ulrich Gergs, Timo Gerigk, Jonas Wittschier, Constanze T. Schmidbaur, Clara Röttger, Mareen Mahnkopf, Hanna Edler, Hartmut Wache, Joachim Neumann

**Affiliations:** Institute for Pharmacology and Toxicology, Medical Faculty, Martin Luther University Halle-Wittenberg, 06097 Halle (Saale), Germany; ulrich.gergs@medizin.uni-halle.de (U.G.); timo.gerigk@t-online.de (T.G.); jonas@wittschier.com (J.W.); c.schmidbaur@web.de (C.T.S.); clara.roettger@yahoo.de (C.R.); mareen.mahnkopf@outlook.de (M.M.); hanna.edler@med.ovgu.de (H.E.); elisabeth.wache@t-online.de (H.W.)

**Keywords:** serotonin, LPS, hypoxia, ischemia, PP2A transgenic mice, inflammation, 5-HT_4_ receptor, transgenic mice, cardiac hypertrophy

## Abstract

The current study aimed to deepen our knowledge on the role of cardiac 5-HT_4_ receptors under pathophysiological conditions. To this end, we used transgenic (TG) mice that overexpressed human 5-HT_4a_ receptors solely in cardiac myocytes (5-HT_4_-TG mice) and their wild-type (WT) littermates that do not have functional cardiac 5-HT_4_ receptors as controls. We found that an inflammation induced by lipopolysaccharide (LPS) was detrimental to cardiac function in both 5-HT_4_-TG and WT mice. In a hypoxia model, isolated left atrial preparations from the 5-HT_4_-TG mice went into contracture faster during hypoxia and recovered slower following hypoxia than the WT mice. Similarly, using isolated perfused hearts, 5-HT_4_-TG mice hearts were more susceptible to ischemia compared to WT hearts. To study the influence of 5-HT_4_ receptors on cardiac hypertrophy, 5-HT_4_-TG mice were crossbred with TG mice overexpressing the catalytic subunit of PP2A in cardiac myocytes (PP2A-TG mice, a model for genetically induced hypertrophy). The cardiac contractility, determined by echocardiography, of the resulting double transgenic mice was attenuated like in the mono-transgenic PP2A-TG and, therefore, largely determined by the overexpression of PP2A. In summary, depending on the kind of stress put upon the animal or isolated tissue, 5-HT_4_ receptor overexpression could be either neutral (genetically induced hypertrophy, sepsis) or possibly detrimental (hypoxia, ischemia) for mechanical function. We suggest that depending on the underlying pathology, the activation or blockade of 5-HT_4_ receptors might offer novel drug therapy options in patients.

## 1. Introduction

Serotonin (5-HT) is taken up by animals in the gastrointestinal tract from food [1]. Baganz and Blakely (2013) reported that about 5% of the total 5-HT in mammals is formed in the brain [2]. Peripheral organs, such as the intestinal wall [3], contain 95% of the total amount of 5-HT in the body [4]; from there, the 5-HT can be taken up by various blood cells and transported mainly by thrombocytes to the heart [5,6]. In addition, 5-HT can also be synthesized in the heart, most notably in cardiomyocytes [7,8].

5-HT can lead to vasoconstriction and subsequent hypertension [1,9,10], although it can also directly affect the cells in the heart. It is also known to increase the force of contraction and, thus, has a positive inotropic effect (PIE) on the atrium and the ventricle of some mammals, including humans [7,11,12,13]. Moreover, 5-HT can increase the heart rate by acting on cells in the sinus node in isolated human preparations [14,15] and isolated pig atrium samples [16]; thus, 5-HT has a positive chronotropic effect (PCE). The effects of 5-HT on the cardiovascular system can be mediated by several receptors [17]. In humans and pigs, the PIE [12,16] and PCE of 5-HT are mediated through 5-HT_4_ receptors. The 5-HT_4_ receptors stimulate forming cAMP in the heart via G proteins [18], enhancing protein phosphorylation [19] and leads to the PIE and PCE in the appropriate cell types.

Mice, which are currently widely used as model species because they are quite amenable to genetic manipulation, do not possess 5-HT_4_ receptors in cardiomyocytes [20,21,22,23]. Therefore, we have previously generated and initially characterized transgenic (TG) mice with cardiac myocyte-specific overexpression of the human 5-HT_4a_ receptor, which is the main 5-HT_4_ receptor isoform in the human heart; these mice are 5-HT_4_-TG mice [20]. We found that 5-HT elicited a PIE in isolated electrically driven atrial preparations from 5-HT_4_-TG mice [21]. In addition, in isolated perfused Langendorff hearts from 5-HT_4_-TG mice, 5-HT increased contractility in living 5-HT_4_-TG mice and induced a PCE in spontaneously beating right atrial preparations and isolated hearts from 5-HT_4_-TG mice. However, none of these effects were present in preparations from their wild-type (WT) littermates [20,21]. The effects of 5-HT in 5-HT_4_-TG mice were accompanied by increased phosphorylation of proteins, such as phospholamban or the troponin inhibitor, which are thought to lead, at least in part, to hastened relaxation of the heart. There was also an increased incidence of arrhythmias in isolated right atrial preparations from 5-HT_4_-TG mice compared with WT mice under basal conditions (i.e., without the addition of drugs). The incidence of arrhythmias was enhanced after applying 5-HT to isolated transgenic atria [23]. The results may be attributed to the known spontaneous release of Ca^2+^ from the sarcoplasmic reticulum into the cytosol in 5-HT_4_-TG mice [20]. It has been suggested that the 5-HT released from damaged thrombocytes might contribute to or prolong the duration of atrial fibrillations in patients [18].

In the present work, we wanted to gain deeper insight into the putative pathophysiological roles of 5-HT_4_ receptors in the heart. We hypothesized that the heart’s response to cardiac stressors like sepsis, hypoxia, ischemia or genetically caused hypertrophy is influenced by serotonin via 5-HT_4_ receptors. Therefore, mice or murine cardiac preparations were subjected to classical stressful interventions with different pathophysiological stimuli and, thus, diverse putative biochemical pathways were activated. These pathways translated into different physiological endpoints. The main finding in this communication was that 5-HT_4_ receptors can be involved in certain cardiac stress responses and should be further studied as a therapeutic treatment for cardiac diseases in humans. Progress reports in the form of abstracts have been previously published [24,25,26,27,28,29,30].

## 2. Materials and Methods

### 2.1. Transgenic Mice

This investigation conformed to the Guide for the Care and Use of Laboratory Animals published by the National Research Council (2011) [31]. Animals were maintained and handled according to the approved protocols of the Animal Welfare Committee of the University of Halle-Wittenberg, Germany. The experimental protocols were approved by the local committee on the ethics of animal experiments of the state Sachsen-Anhalt (Permit Number: 42502-02-691 MLU and 42502-2-1537 MLU). Echocardiography was performed under isoflurane anesthesia, and for euthanasia of mice, pentobarbital (50 mg/kg body weight i.p.) was used. The generation and basal characterization of 5-HT_4_-TG mice with cardiac expression of the human 5-HT_4_ receptor and mice overexpressing the catalytic subunit of PP2A in the heart (PP2A-TG) were reported previously in detail [20,32]. Immunohistology demonstrated localization of the 5-HT_4_ receptor with the *t*-tubule system in 5-HT_4_-TG cardiomyocytes [20], whereas overexpressed PP2A was evenly distributed throughout the cardiomyocytes in PP2A-TG hearts [32]. Both TG mouse strains (CD1 background) expressed the transgene under control of the mouse α-myosin heavy chain promoter, specifically in cardiac myocytes of the atrium and ventricle [20,32]. In all experimental groups except the mice in the experimental setup with the double transgenic mice, we used 150-day-old mice with equal distribution of both sexes. In all experimental groups for the genetically induced hypertrophy (WT, mono-transgenic, double transgenic), we used 12 months old mice because the hypertrophy induced by PP2A overexpression in PP2A-TG mice was an age-dependent process.

### 2.2. Contraction Studies of the Atrium

The contraction studies of the atrium followed our previously published procedures [20,33,34]. In brief, hearts were excised from anesthetized mice (50 mg/kg pentobarbital i.p.); left and right atria were prepared and transferred to 10 mL organ baths containing the following physiological buffer that was adjusted to pH 7.4 at 37 °C (in mM): NaCl 119.8, KCl 5.4, CaCl_2_ 1.8, MgCl_2_ 1.05, NaH_2_PO_4_ 0.42, NaHCO_3_ 22.6, Na_2_EDTA 0.05, ascorbic acid 0.28 and glucose 5.0, continuously gassed with Carbogen (95% O_2_, 5% CO_2_). Left atria were electrically stimulated (1 Hz), whereas right atria contracted spontaneously. The contractions of the atrial preparations were detected by isometric force transducers, and the data were recorded using a PowerLab system (ADInstruments, Oxford, UK). Hypoxia was induced by switching the gas supply of the organ bath from carbogen to a mixture of 95% N_2_ and 5% CO_2_ (Linde Gas Supply Company, Pullach, Germany). After 20 min, the gas supply was switched back to carbogen (as reported [33]). In some experiments, different protocols were used, including hypoxic preconditioning. Details are provided in the appropriate legends and figures.

### 2.3. Contraction Studies of the Ventricle (Langendorff Procedure)

The mouse hearts were prepared using the Langendorff procedure as previously described [33,35,36]. In brief, hearts were excised from anesthetized and heparin-treated mice (50 mg/kg pentobarbital and 1.5 units heparin i.p.). Hearts were fixed via the aorta to a 20-gauge cannula, and retrogradely perfused with a constant flow of 2 mL/min on a Langendorff apparatus with modified Krebs-Henseleit buffer (37 °C, pH 7.4) containing (in mM): NaCl 118, NaHCO_3_ 25, Na-EDTA 0.5, KCl 4.7, KH_2_PO_4_, 1.2, MgSO_4_, 1.2, CaCl_2_ 2.5 and glucose 11, continuously gassed with Carbogen (95% O_2_, 5% CO_2_). The force of contraction of the heart was detected by an isometric force transducer attached to the apex of the heart, and the data were recorded using a PowerLab system (ADInstruments, Oxford, UK). From these data, the beating rate, the first derivative of left ventricular force (+dF/dt and–dF/dt) and other time parameters were calculated. No-flow ischemia was begun by stopping the peristaltic pump of the buffer for 20 min. Reperfusion was brought about by restarting the pump (as reported [33]). Drugs were applied by a syringe pump (B. Braun, Melsungen, Germany) attached to the system, and when the maximum effect was reached (after 5 min), the hearts were quick-frozen in liquid nitrogen and stored at −80 °C until further use.

### 2.4. Quantitative Polymerase Chain Reaction (qPCR)

Quantitative PCR was done with slight modifications of the standard procedures in our lab [33,37,38]. The abundance of mRNAs of typical marker genes was assessed. Therefore, total RNA was isolated from homogenized frozen tissue using TRIzol^®^ reagent (Ambion Life Technologies, Carlsbad, CA, US) following the manufacturer’s instructions. It was reverse transcribed using the maxima first-strand cDNA synthesis kit for RT–qPCR with dsDNase (Thermo Fisher Scientific, Dreieich, Germany). Real-time PCR amplification was performed in the CFX Connect real-time PCR detection system (Bio-Rad, Feldkirchen, Germany) using iTaq™ Universal SYBR^®^ green supermix (Bio-Rad, Feldkirchen, Germany), 10 ng of cDNA and 500 nM of each of the primers. Melting curve analysis was performed to monitor the amplification products. The relative mRNA values were calculated using the 2^–∆∆CT^ method by Livak and Schmittgen (2001) and normalized to expressing the housekeeping gene GAPDH [39]. Following primers were used (5′–3′): GAPDH-forward, ATGCATCCTGCACCACCAAC; GAPDH-reverse, ATGCCTGCTTCACCACCTTC; ANP-forward, gtgcggtgccaacacagat; ANP-reverse, gcttcctcagtctgctcactca; BNP-forward, ccagtctccagagcaattcaa; BNP-reverse, agctgtctctgggccatttc; NFκB1-forward, GAAATTCCTGATCCAGACAAAAAC; NFκB1-reverse, ATCACTTCAATGGCCTCTGTGTAG; IκBα-forward, ATGAAGGACGAGGAGTACGAGCAA; IκBα-reverse, TCTCTTCGTGGATGATTGCCAA; IL-1β-forward, TCGTGCTGTCGGACCCATAT; IL-1β-reverse, GTCGTTGCTTGGTTCTCCTTGT; IL-6-forward, CCGGAGAGGAGACTTCACAG; IL-6-reverse, TTCTGCAAGTGCATCATCGT; TNFα-forward, CACACTCAGATCATCTTCTCAAAA; TNFα-reverse, GTAGACAAGGTACAACCCATCG; LBP-forward, AGATCACACTACCGGACTTCAGCG; LBP-reverse, TTCCATTTGCCTCGGACACCGATG; TLR4-forward, CTCTGCCTTCACTACAGAGAC; TLR4-reverse, TGGATGATGTTGGCAGCAATG; human 5-HT_4_R-forward, GTTGAACCCTTTTCTCTACG; human 5-HT_4_R-reverse, TTTCTCGAGTTCCTGATGAT.

### 2.5. Western Blotting

Homogenates from atrial and ventricular tissue samples were prepared as described in previous studies [33,34,40] in 300 µL of 10 mM NaHCO_3_ with 100 µL of 20% SDS. Crude extracts were incubated at 25 °C for 30 min before centrifugation to remove debris. The supernatants, or homogenates, were then separated and stored at −80 °C until further use. Western blot analysis was performed as previously described [20,32,33]. Briefly, aliquots of 60 µg of protein were loaded per lane. Bands were detected using enhanced chemifluorescence (ECF, GE Healthcare Europe, Freiburg, Germany) with a Typhoon 9410 variable mode imager (GE Healthcare Europe). The following primary antibodies were used in this study: rabbit polyclonal anti-calsequestrin (1:1000; #SP5340P, Acris Antibodies, Herford, Germany), mouse monoclonal anti-PLB (1:2000; #A010-14, Badrilla, Leeds, UK), rabbit polyclonal anti-phospho-PLB (1:5000; #A010-12 and #A010-13, Badrilla, Leeds, UK), rabbit monoclonal anti-p44/42 MAPK and anti-phospho-p44/42 MAPK (1:1000; #4695 and #4370, Cell Signaling Technology Europe, Leiden, The Netherlands), rabbit polyclonal anti-p38 MAPK and rabbit monoclonal anti-phospho-p38 MAPK (1:1000; #9212 and #4511, Cell Signaling Technology Europe, Leiden, The Netherlands), rabbit monoclonal anti-PP2Ac (1:1000; #ab32141, Abcam, Berlin, Germany), and mouse monoclonal anti-SERCA (1:1000; kindly provided by L.R. Jones, Indianapolis, IN, USA). Note that the rabbit polyclonal anti-phospho-PLB antibodies were raised against PLB-peptide phosphorylated at serine 16 or threonine 17. The characteristics and use of these antibodies have been previously reported by our group [38,41]. Unedited Western blots can be found in the supplementary figures.

### 2.6. Echocardiography

Echocardiography in spontaneously breathing mice was performed under anesthesia with 1.5% isoflurane. The procedure was previously described [20,33]. In brief, echocardiographic measurements were performed using a Vevo 2100 system equipped with an MS 550D transducer (Visual Sonics, Toronto, Canada). The anesthetized mice were fixed on a 37 °C heating pad, and two-dimensional images and M-mode tracings from the parasternal long-axis view and short-axis view were recorded. Moreover, pulsed wave Doppler measurements and tissue Doppler imaging were performed. If applicable, isoproterenol or serotonin (100 µL of 1 mM solution) was injected where indicated.

### 2.7. Lipopolysaccharide Treatment

Mice were IP-injected with 30 µg/g body weight of lipopolysaccharide (LPS; O55:B5 from *E. coli*), which was dissolved in isotonic NaCl solution. The control mice were injected with isotonic NaCl solution [37]. Cardiac function was measured by echocardiography before LPS (NaCl) application (pre-drug values = basal values) and seven hours after LPS (NaCl) application. Thereafter, the hearts were excised from the still anesthetized mice for further analyses.

### 2.8. Data Analysis

Data shown are means ± SEM. Statistical significance was estimated by analysis of variance (ANOVA) followed by a Bonferroni post-test or by a Student’s *t*-test as appropriate. A *p*-value < 0.05 was considered to be significant. For statistical analysis and data presentation, the software GraphPad Prism 5.0 (GraphPad Software, San Diego, California, USA) was used.

### 2.9. Drugs and Materials

All chemicals were of analytical grade. LPS (#L2880), serotonin and isoproterenol were purchased from Sigma-Aldrich (Munich, Germany).

## 3. Results

### 3.1. Cardiac Response to LPS-induced Sepsis

**Figure 1:**
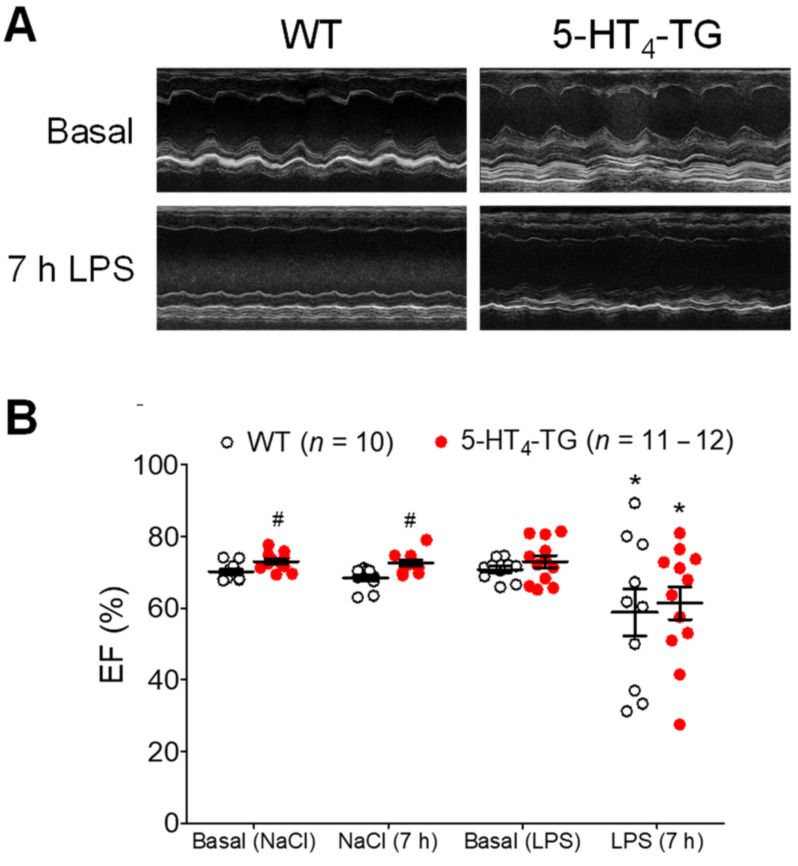
Echocardiography of LPS-treated mice. (**A**) M-mode pictures of WT and 5-HT_4_-TG, basal and 7 h after LPS treatment. (**B**) LPS treatment (7 h) led to a deterioration of cardiac function demonstrated as decreased left ventricular ejection fraction (EF). Number in brackets indicates the number of mice studied. WT = wild-type mice, 5-HT_4_-TG=5-HT_4_-transgenic mice. Data shown are means ± SEM. * *p* < 0.05 vs. basal; ^#^ *p* < 0.05 vs. WT.

**Figure 2:**
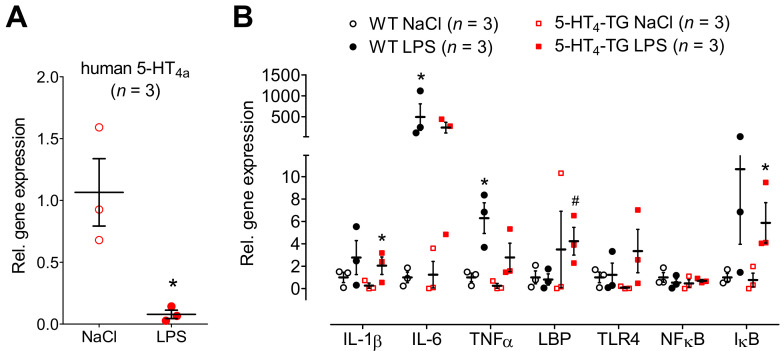
mRNA expression in WT and 5-HT_4_-TG mice, treated either with LPS or NaCl. (**A**) The mRNA coding for the overexpressed human 5-HT_4_-receptor was greatly downregulated in hearts of TG after LPS treatment. Ordinate: mRNA expression normalized to GAPDH expression. Three mice were studied in each genotype. * *p* < 0.05 vs. NaCl. (**B**) LPS-induced heart failure was accompanied by increased mRNA expression of cytokines like interleukin 1 β and 6 (IL-1 β, IL-6) and tumor necrosis factor α (TNFα) in both 5-HT_4_-TG and WT. The mRNA of the LPS-binding protein (LBP) and the Toll-like receptor 4 (TLR4) was increased in 5-HT_4_-TG, but not in WT. Whereas the mRNA of NFκB was unchanged, the mRNA of IκBα was to a similar extent increased by LPS in WT and 5-HT_4_-TG. Three mice were studied in each group, and injection of NaCl served as control. WT = wild-type mice, 5-HT_4_-TG = 5-HT_4_-transgenic mice. Data shown are means ± SEM. * *p* < 0.05 vs. NaCl; ^#^ *p* < 0.05 vs. WT.

Initially, we wanted to simulate the cardiovascular burden of sepsis on mice. To this end, the animals were treated with LPS. As expected, the LPS deteriorated cardiac performance in a time-dependent way. For instance, the left ventricle ejection fraction (EF) was reduced, as evidenced when the typical M-mode echocardiographs of the basal contraction in WT mice and 5-HT_4_-TG mice (Figure 1A, Ctr) were compared with the echocardiographs after 7 h of LPS treatment (Figure 1A, LPS). After summarizing the results of several experiments, it was apparent that an injection of LPS diminished cardiac function using EF as a parameter of left ventricular contractility compared with the effect of a control injection of isotonic NaCl solution (Figure 1B). However, there was no difference in the detrimental effect of LPS on the EF between the WT mice and 5-HT_4_-TG mice. Similarly, LPS treatment led to reduced blood flow through the ascending and descending aortae, pulmonary arteries, pulmonary veins and superior vena cava; however, there was no difference between the 5-HT_4_-TG mice and WT mice (Supplementary Materials Table S1). When we observed the E-wave and the A-wave through the mitral valve to assess the diastolic function of the heart, we determined a time-dependent decrease for the E-wave and A-wave after LPS treatment in both 5-HT_4_-TG and WT mice, but the ratio of E and A was unchanged after LPS (Supplementary Materials Figure S1). A similar pattern was noted concerning the E-wave and the A-wave through the tricuspid valve (Supplementary Materials Figure S1). Interestingly, we noticed a substantial decline in expressing mRNA for the transgenic 5-HT_4a_ receptor in the heart of the 5-HT_4_-TG mice after application of LPS (Figure 2A). Other genes measured were also regulated. LPS elevated the mRNA expression, at least partially, but apparently not differentially in the hearts of the 5-HT_4_-TG and WT mice. This is shown in Figure 2B for the interleukins IL-1β and IL-6, as well as for tumor necrosis factor α (TNFα), LPS-binding protein (LBP), Toll-like receptor 4 (TLR4), nuclear factor κB (NFκB) and inhibitor of NFκB (IκB), which are typical parameters that are elevated in inflammation.

### 3.2. Atrial Response to Hypoxia in Vitro

**Figure 3:**
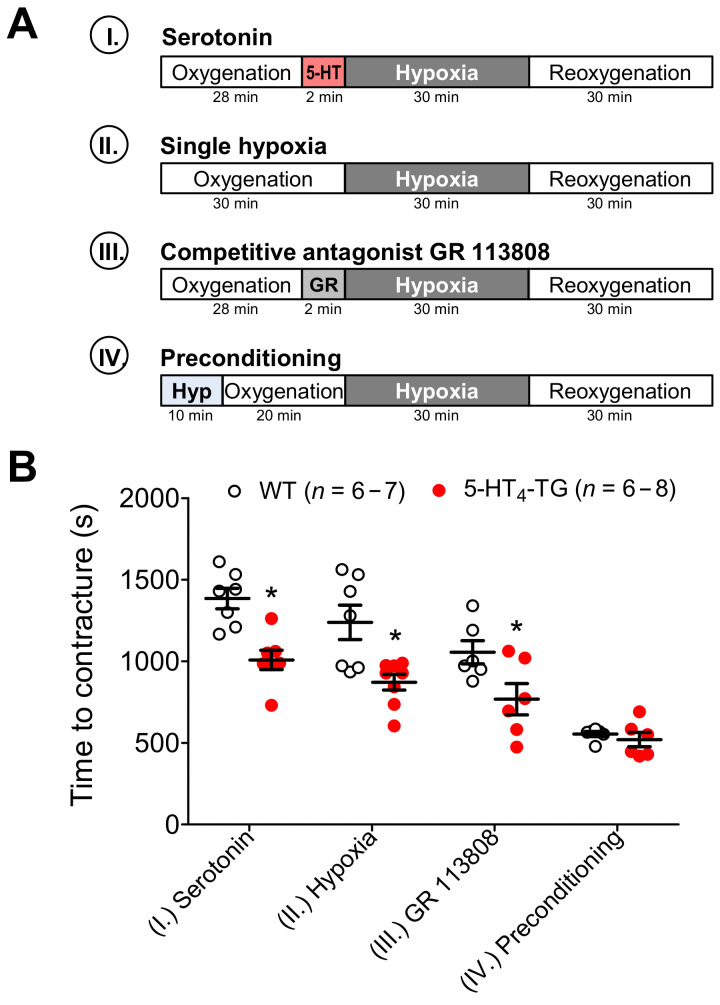
Hypoxia in atrial preparations. (**A**) The scheme demonstrates the experimental protocols of the experiments. Paced left atrial preparations from wild-type mice (WT), or 5-HT_4_-transgenic mice (5-HT_4_-TG) were allowed to equilibrate in the organ bath in buffer saturated with carbogen (=oxygenation, 5% CO_2_ and 95% O_2_). Then as indicated, four protocols were performed: (I.) 28 min of oxygenation followed by addition of serotonin (5-HT, 1 µM) for 2 min; (II.) 30 min of oxygenation; (III.) 28 min of oxygenation followed by addition of the 5-HT_4_-antagonist GR 113808 (GR, 1 µM) for 2 min; (IV.) 10 min of hypoxia (Hyp) followed by 20 min of oxygenation. Thereafter, all conditions (I.–IV.) include the same procedure: 30 min of hypoxia (5% CO_2_ and 95% N_2_) and then again carbogen (reoxygenation). (**B**) During hypoxia, left atrial preparations lose their ability to completely relax, and an increase in diastolic tension (contractures) occurs. Under the setups serotonin (1 µM), single hypoxia and GR 113808 (1 µM), 5-HT_4_-TG atria developed contractures earlier than WT atria. Roman numbers indicate the experimental protocol, and numbers at the bottom of the columns indicate the number of experiments. Data shown are means ± SEM. * *p* < 0.05 vs. WT.

**Figure 4:**
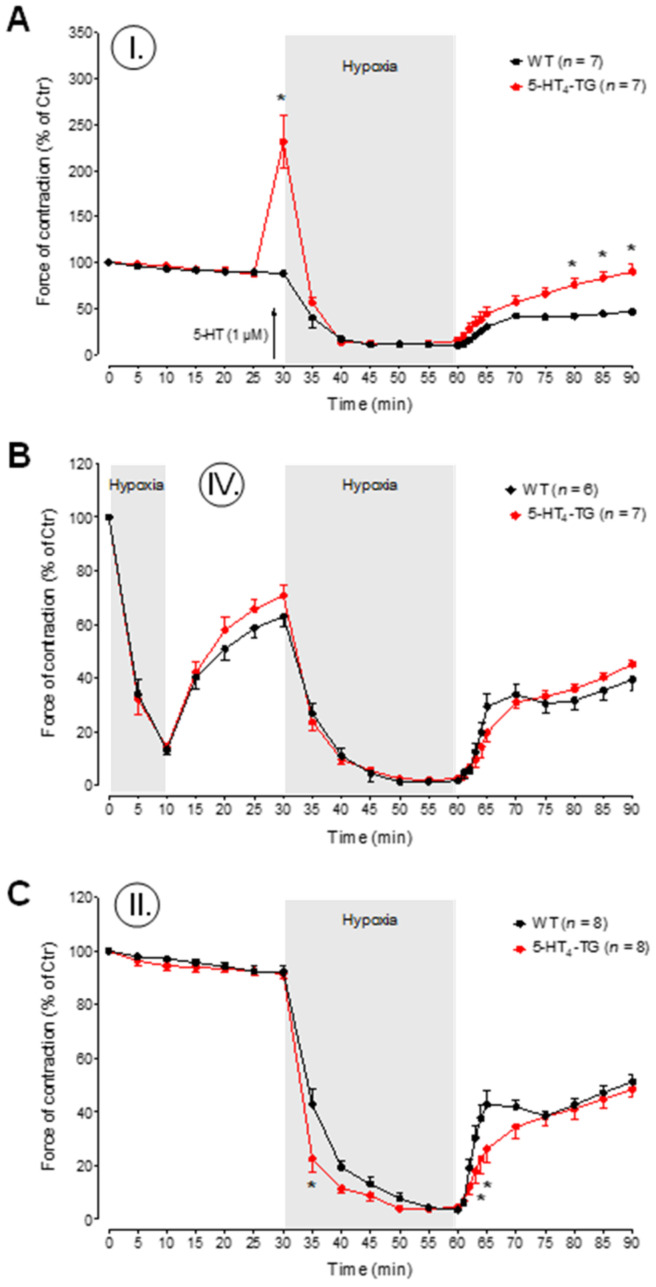
Time course of hypoxia in left atrial preparations. (**A**–**C**) The force of contraction in% of control (Ctr = initial force at the beginning of the experiment) during the time of oxygenation and hypoxia is presented. After the addition of serotonin (5-HT, 1 μM, protocol I), the relative force was greatly increased in 5-HT_4_-TG and reached initial values again after hypoxia and reoxygenation (**A**). Preconditioning (protocol IV) as short hypoxia for 10 min was not beneficial (**B**). Under the condition of single hypoxia (protocol II), force decline was faster in TG left atria than in WT (**C**). WT = wild-type mice, 5-HT_4_-TG=5-HT_4_-transgenic mice. Data shown are means ± SEM. * *p* < 0.05 vs. WT.

Under the studied hypoxic conditions, I-III (see the schematic protocols in Figure 3A), the left atria from the 5-HT_4_-TG mice went into contracture faster than the left atria from the WT mice (Figure 3B). Following the hypoxic preconditioning protocol IV, the time to contracture was no longer different between WT and 5-HT_4_-TG left atria (Figure 3B). To determine the force of contraction of heart muscle under hypoxic conditions, 5-HT was given right before the initiation of hypoxia and was found to exert a PIE in 5-HT_4_ TG mice, but not in WT mice (Figure 4A). However, in 5-HT_4_ TG mice treated with 5-HT in the organ bath, under hypoxic conditions, the force of heart muscle contraction gradually decreased to similar levels of the WT mice (Figure 4A). Upon reoxygenation, the force of contraction in these mice gradually increased to higher levels than in the WT mice, although they did not reach the value before hypoxia (Figure 4A). A similar pattern was noted in the beating rate of the right atrial preparations (Supplementary Materials Figure S2). There was no difference between the WT mice and the 5-HT_4_-TG mice (Figure 4B) concerning the effect of preconditioning (Figure 3A, protocol IV). Using a typical protocol for the single preconditioning of mice, there was no difference between the WT mice and 5-HT_4_-TG mice during the initial phase of reoxygenation or the second phase of reoxygenation (Figure 4B). Repeating the same protocol as used in Figure 4A, but with the absence of external 5-HT in the organ bath (protocol II), the 5-HT_4_-TG mice had lower levels of the force of contraction after 1 min and 2 min of hypoxia and the force increased more slowly than in WT mice upon reoxygenation (Figure 4C).

### 3.3. Ventricular Response to Ischemia In Vitro

**Figure 5:**
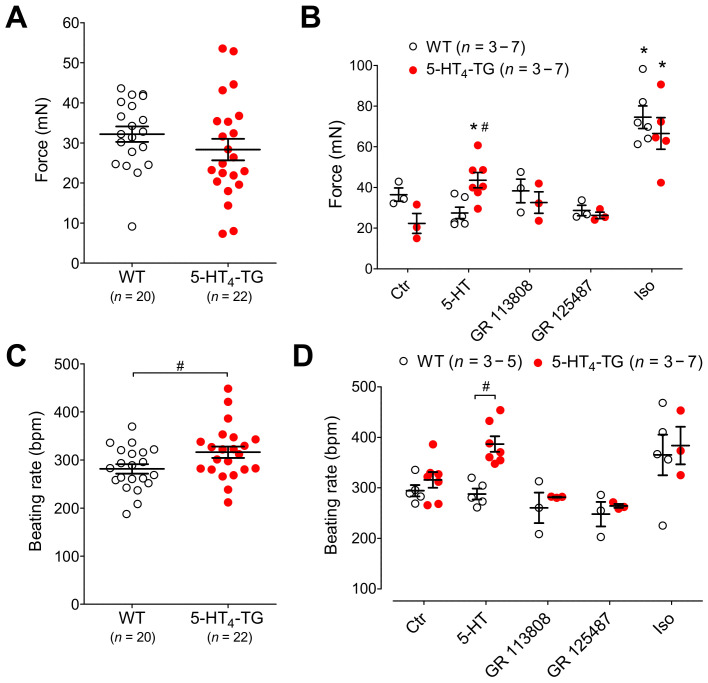
Basal characteristics of isolated perfused heart preparations from WT and 5-HT_4_-TG mice. (**A**) Basal force of contraction in mN. (**B**) Effects of agonists and antagonists on the force of contraction after 5 min (maximum was reached). (**C**) Basal beating rate in beats per minute (bpm). (**D**) Effects of agonists and antagonists on the beating rate after 5 min. Ctr, control, Iso, isoproterenol (1 µM), 5-HT, serotonin (1 µM) and 5-HT (1 µM) in the presence of the 5-HT_4_-receptor antagonist’s GR 113808 (1 µM) or GR 125487 (1 µM). WT = wild-type mice, 5-HT_4_-TG = 5-HT_4_-transgenic mice. Data shown are means ± SEM. * *p* < 0.05 vs. Ctr, ^#^ *p* < 0.05 vs. WT.

**Figure 6:**
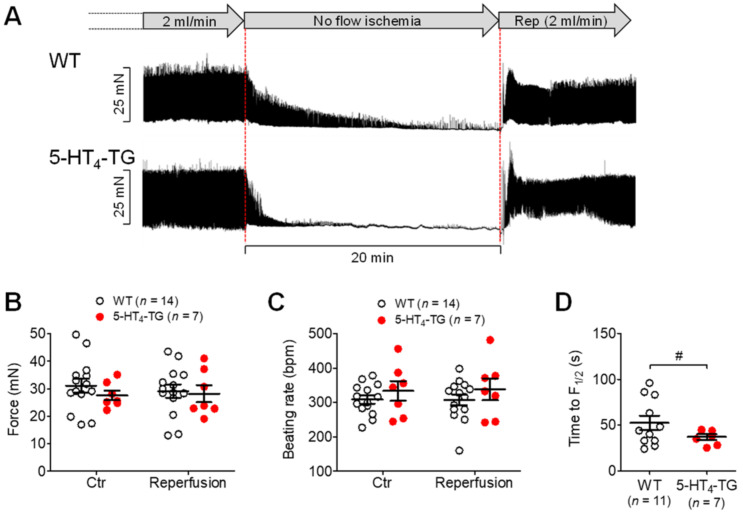
Ischemia and reperfusion in isolated perfused heart preparations. (**A**) Exemplary recordings of the time course of force reduction (ischemia) and force recovery (reperfusion, Rep) in isolated perfused heart preparations from WT and 5-HT_4_-TG. The perfusion rate was always 2 mL/min. No flow ischemia indicates global ischemia of the heart by stopping the perfusion pump. Horizontal bar: 20 min of ischemia. A period of 20 min ischemia did not cause permanent damage because, after reperfusion, force (**B**) and heart rate (**C**) of both 5-HT_4_-TG and WT reached preischemic values again. Time to 50% decline of developed force (F_½_) during ischemia was reduced in 5-HT_4_-TG compared to WT (**D**). WT = wild-type mice, 5-HT_4_-TG=5-HT_4_-transgenic mice. Data shown are means ± SEM. ^#^ *p* < 0.05 vs. WT.

In isolated spontaneously beating Langendorff hearts, under normoxic or control conditions, the basal developed force was comparable in the left ventricle, although the beating rate was somewhat higher in the 5-HT_4_-TG mice than WT mice (Figure 5A,C). When 1 µM β-adrenoceptor agonist isoproterenol was given, it increased the basal developed force to a comparable extent in both the WT mice and the 5-HT_4_-TG mice (Figure 5B). When 5-HT was given, it only increased the basal developed force in the 5-HT_4_-TG mice and not in the WT mice, as expected, since WT cardiomyocytes do not express functional 5-HT_4_ receptors; the increase induced by 5-HT in the 5-HT_4_-TG mice was blocked by either the specific 5-HT_4_ receptor antagonist GR 113808 or GR 125487 (Figure 5B). Moreover, 5-HT increased the beating rate only in the 5-HT_4_-TG mice and not in the WT mice (Figure 5D). The maximum effects of the agonists (5-HT and isoproterenol) were reached about five minutes after starting the syringe pump, and therefore, the effects of all drugs mentioned above were recorded five minutes after starting drug perfusion. Under conditions of global ischemia, the force declined faster in the 5-HT_4_-TG mice than in WT mice (Figure 6A). It should be noted that the basal developed force withstood ischemia very well because there was no difference between the force and the beating rate before hypoxia and after complete reoxygenation (Figure 6B,C). Like hypoxic atria, the time to 50% decline of developed force (F_½_) during ischemia was reduced in 5-HT_4_-TG compared to WT (Figure 6D).

**Figure 7:**
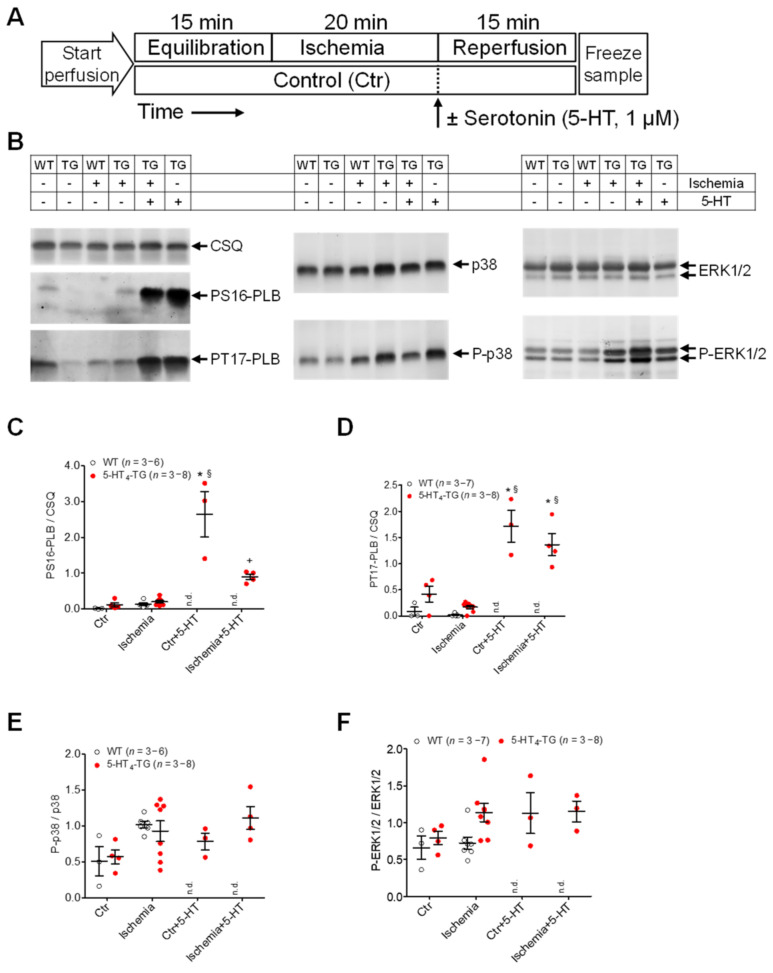
Protein phosphorylation after ischemia/reperfusion in isolated perfused hearts of WT and 5-HT_4_-TG mice. (**A**) The scheme demonstrates the protocols (Langendorff perfusion: 2 mL/min flow): (1) 15 min equilibration, 20 min ischemia by stopping the perfusion followed by 15 min reperfusion or 50 min continuous perfusion with saline buffer as time control; (2) 15 min equilibration, 20 min ischemia and 15 min reperfusion in the presence of 1 µM serotonin (5-HT) or 35 min perfusion followed by 15 min perfusion with 5-HT (1 µM) as time control without ischemia. (**B**) Representative Western blots. The loading scheme is shown in the table above the blots. TG = 5-HT_4_-TG. (**C**) Phosphorylation of phospholamban at serine-16 (PS16-PLB) and (**D**) threonine-17 (PT17-PLB) normalized to cardiac calsequestrin (CSQ). (**E**) Phosphorylation of the mitogen-activated protein kinases (MAPK) p38 and (**F**) ERK1/2 normalized to the non-phosphorylated MAPKs. Ordinates: Ratio of phosphoproteins to calsequestrin or non-phosphorylated MAPKs in arbitrary imager units. Data shown are means ± SEM. * *p* < 0.05 vs. Ctr; ^§^ *p* < 0.05 vs. ischemia; ^+^ *p* < 0.05 vs. Ctr + 5-HT. WT = wild-type mice, 5-HT_4_-TG = 5-HT_4_-transgenic mice; n.d., not determined (As WT preparations did not respond to 5-HT, perfusion with 5-HT was exclusively done with 5-HT_4_-TG hearts).

After the perfused hearts were freeze-clamped as depicted in the appropriate protocols (Figure 7A), Western blot analysis was performed on thawed and homogenized samples (representative Western blots are shown in Figure 7B). The following alterations in protein expression were observed: ischemia followed by an application of 5-HT increased phospholamban phosphorylation in 5-HT_4_-TG mice at serine 16 and threonine 17, although the effect was less than under normoxic conditions (Figure 7C,D). It was noteworthy that ischemia depressed 5-HT-induced phosphorylation at serine 16 but not 5-HT-induced phosphorylation at threonine 17 (Figure 7C,D). Ischemia itself or applying 5-HT led to an increased phosphorylation state of p-38 in 5-HT_4_-TG mice and WT mice (Figure 7E). It should be noted that even under normoxic conditions and after ischemia, 5-HT elevated the phosphorylation state of p-ERK1/2 (Figure 7F). It should be noted that the experiments with the 5-HT application were performed only with transgenic hearts because from previous studies [20], it is known that WT hearts did not respond to 5-HT, including the phosphorylation of PLB. Under basal perfusion conditions (see the scheme Figure 7A), the mRNA of ANP and BNP was more highly expressed in the 5-HT_4_-TG mice than in the WT mice, although the mRNA decreased to similar levels upon ischemia (Supplementary Materials Figure S3). A similar pattern was seen with the mRNA coding for IL-6, IL-1β and IκB (Supplementary Materials Figure S3).

### 3.4. Influence of 5-HT_4_ Receptors on a Genetically Induced Hypertrophy

**Figure 8:**
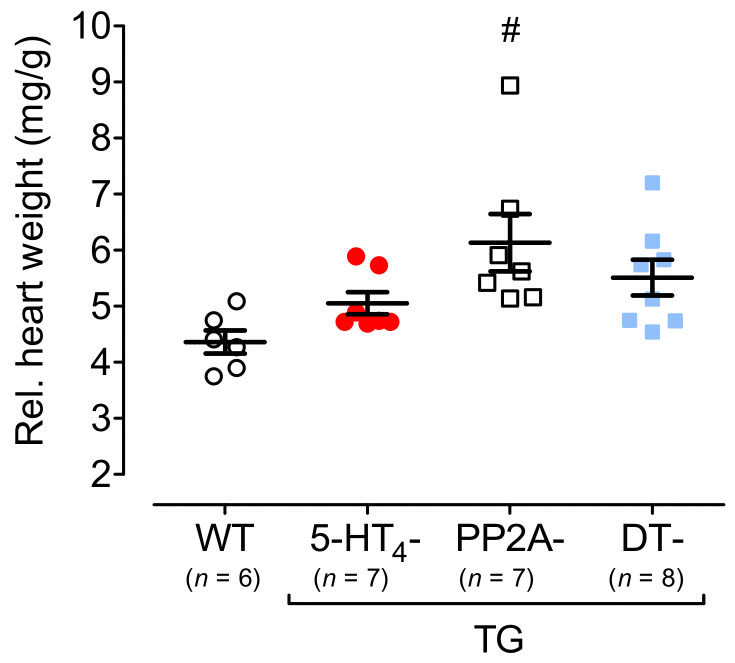
Heart weight. Relative heart weights of 5-HT_4_-TG, PP2A-TG and double transgenic (DT) mice at 12 months of age compared to wild-type (WT) mice. Ordinate: heart weight in milligrams (mg) divided by body weight in grams (g). TG, transgenic mice. Numbers in brackets indicate the numbers of mice studied. Data shown are means ± SEM. ^#^ *p* < 0.05 vs. WT.

**Figure 9:**
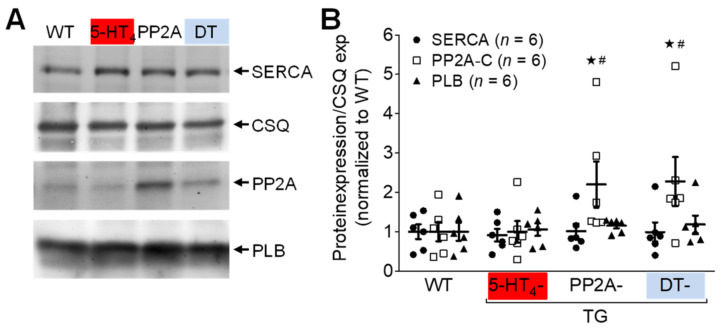
Protein expression in double transgenic mice. Protein expression of SERCA, PP2A, PLB and CSQ in hearts of wild-type (WT), 5-HT_4_-TG, PP2A-TG and double transgenic (DT) mice. (**A**) Representative Western blots. (**B**) Quantification of ventricular proteins. Data were normalized to CSQ (loading control) and to mean WT expression. TG, transgenic mice. Data shown are means ± SEM. ^#^ *p* < 0.05 vs. WT; ^★^ *p* < 0.05 vs. 5-HT_4_-TG.

To study hypertrophy, 5-HT_4_-TG mice were crossbred with PP2A-TG mice (mice with cardiac overexpression of the catalytic subunit of PP2A) first described in Gergs et al. 2004 [32] to produce PP2A-TG × 5-HT_4_-TG mice (DT mice). The PP2A-TG mice, 5-HT_4_-TG mice and DT mice exhibited increases in the relative heart weight (Figure 8). The body weights were similar in all groups (Supplementary Materials Table S2). The overexpression of the catalytic subunit of PP2A in the PP2A-TG mice became apparent by Western blots of PP2A-TG and DT hearts (Figure 9A) and is summarized in Figure 9B. In addition, expressing calsequestrin, the sarcoplasmic reticulum Ca^2+^ ATPase (SERCA) and phospholamban (PLB) were measured and found to be unaltered between genotypes (Figure 9). Unfortunately, these kinds of samples were not suitable to measure the phosphorylation of PLB, namely because the isolation procedure of the hearts could not be controlled as well as for isolated perfused hearts.

**Figure 10:**
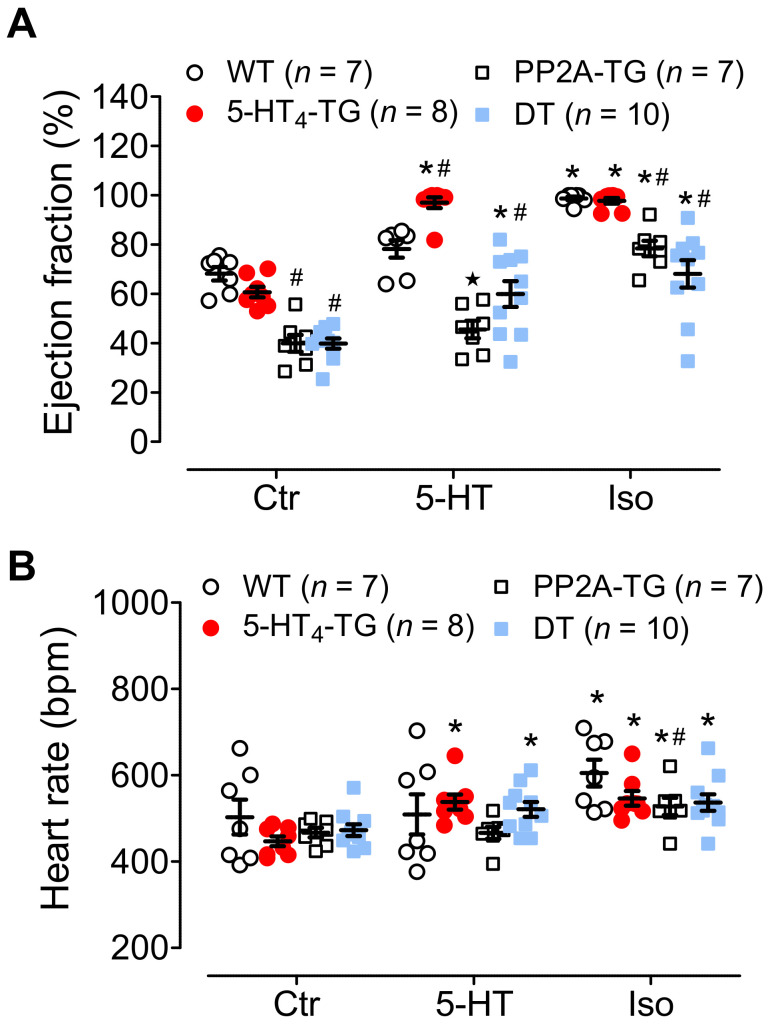
Echocardiography of double transgenic mice. Echocardiography of wild-type (WT), 5-HT_4_-transgenic (5-HT_4_-TG), PP2A-transgenic (PP2A-TG) and double transgenic (DT) mice. (**A**) Basal ejection fraction (Ctr) was reduced in PP2A-TG and DT mice, and 5-HT increased EF only in 5-HT_4_-TG and DT mice. β-adrenergic stimulation by isoproterenol (Iso) increased EF less in PP2A-TG and DT compared to the other groups. (**B**) Basal heart rate (Ctr) was not different between genotypes, and positive chronotropic effects of 5-HT were only noted in 5-HT_4_-TG and DT mice. However, β-adrenergic stimulation (Iso) increased heart rate in all groups. Numbers in bars indicate the numbers of mice studied. Data shown are means ± SEM. * *p* < 0.05 vs. Ctr; ^#^ *p* < 0.05 vs. WT.

**Figure 11:**
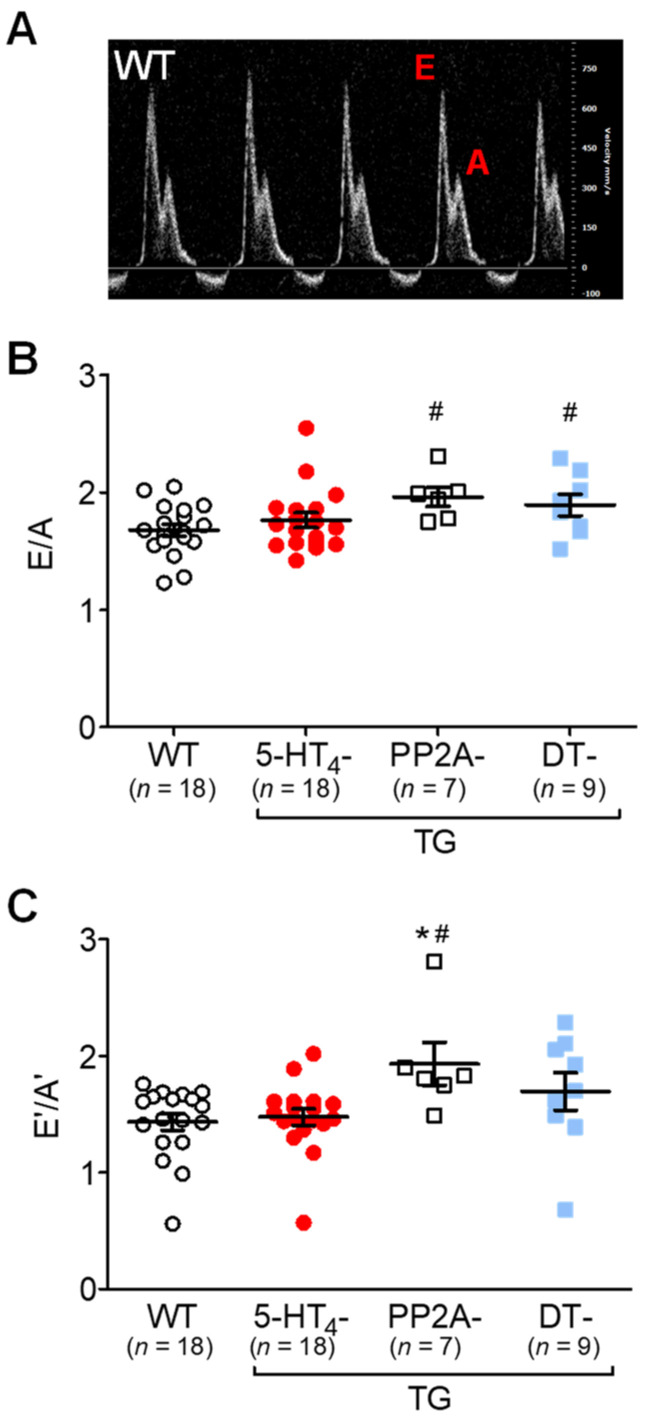
Doppler echocardiography of double transgenic mice. Pulsed wave (PW) Doppler echocardiography of wild-type (WT), 5-HT_4_-transgenic (5-HT_4_-TG), PP2A-transgenic (PP2A-TG) and double transgenic (DT) mice. (**A**) A typical pattern of E wave and A wave in mitral flow. The E wave represents the early filling of the ventricle. The A wave represents the atrial contraction. (**B**) E divided by A was increased in PP2A-TG and in DT. (**C**) By tissue Doppler imaging of the left ventricular posterior wall, the early (E’) and late (A’) diastolic and systolic maximum tissue velocity was assessed. The E’ wave corresponds to the motion of the posterior wall during early diastolic filling of the left ventricle, and the A’ wave originates from atrial contraction during the late filling of the left ventricle. An increased E’/A’ quotient was noted in PP2A-TG but not in DT mice. Numbers in bars indicate the numbers of mice studied. Data shown are means ± SEM. ^#^ *p* < 0.05 vs. WT; ^★^ *p* < 0.05 vs. 5-HT_4_-TG.

Cardiac function was measured in vivo using echocardiography. Under unstimulated conditions, the EF was smaller in the PP2A-TG mice and DT mice than in the 5-HT_4_-TG mice and WT mice (Figure 10A, Ctr). An IP injection of 5-HT only increased the EF in the 5-HT_4_-TG mice versus the WT mice and in the DT mice versus the PP2A-TG mice (Figure 10A). A similar pattern was noted concerning the beating rate (Figure 10B). For comparison, we studied the contractile response to an IP injection of the β-adrenoceptor agonist isoproterenol. We found that the isoproterenol increased the EF in all genotypes (Figure 10B), although the effect was less in the PP2A-TG mice and DT mice. It should be noted that the basal EF was already diminished in the PP2A-TG mice and DT mice (Figure 10B, Ctr). A similar pattern to that shown in Figure 10A for EF was seen for the beating rate in the same mice (Figure 10B). As labeled in the original recording in Figure 11A, the ratio of E-waves and A-waves is plotted and shown in Figure 11B. It can be seen that the PP2A-TG and DT mice exhibited an elevated ratio compared with the other genotypes (Figure 11B), but the ratio of E’-waves and A’-waves determined by tissue Doppler of the left ventricular posterior wall was elevated only in PP2A-TG mice compared with all other genotypes (Figure 11C). The echocardiographic parameters that were measured are summarized in the Supplementary Materials Table S3.

## 4. Discussion

In the present work, we studied the effect of various stressors like LPS simulating sepsis or oxygen depletion via cardiac hypoxia and ischemia on cardiac function in vitro and in vivo in 5-HT_4_-TG mice. By crossbreeding with PP2A-TG mice, we studied the influence of 5-HT_4_ receptors on a genetically induced hypertrophy. The 5-HT_4_-TG mice had a cardiomyocyte-specific overexpression of the human 5-HT_4_ receptor [20]. The effects on the 5-HT_4_-TG mice were compared with the effects on WT mice without any functional 5-HT_4_ receptors under appropriate control conditions. For all experiments, we randomized for gender, although it is known that there are gender differences in cardiac pathology [42,43]. However, this subject was beyond the scope of the present study. Moreover, we have not yet seen any gender differences in cardiac function in our transgenic mouse models [20,21,22,32].

### 4.1. Lipopolysaccharide-Simulated Sepsis

Studies have shown that plasma 5-HT levels increase after sepsis in human patients. The levels also increase in animal models after experimental induction of sepsis using endotoxins, such as LPS [44]. We do not know whether increased 5-HT levels in plasma would be accompanied by increased mortality, as this was not within the scope of our studies.

Sepsis is mediated in part by the NFκB-mediated activation of genes relevant to inflammatory pathways leading to the release of proinflammatory cytokines [45]. These cytokines were also increased by LPS in our study. A link to serotonin signaling was shown when cecal perforation was used to induce sepsis in rats, and researchers found that the concentration of serotonin in left ventricular cardiomyocytes increased [46]. In septic rats, the expression level of IL-6 also increased [47]. Interestingly, mice with a tryptophan hydroxylase one knockout (TPH1-KO)—TPH1 is the rate-limiting enzyme for the endogenous production of 5-HT in the periphery—that have low levels of 5-HT in the heart, exhibited lower mortality than WT mice with sepsis. These results could possibly mean that 5-HT aggravates sepsis [48]. Using this model, researchers noted increased expression of IL-6 and TNFα in the heart of septic mice, which agreed with our findings [48].

**Figure 12:**
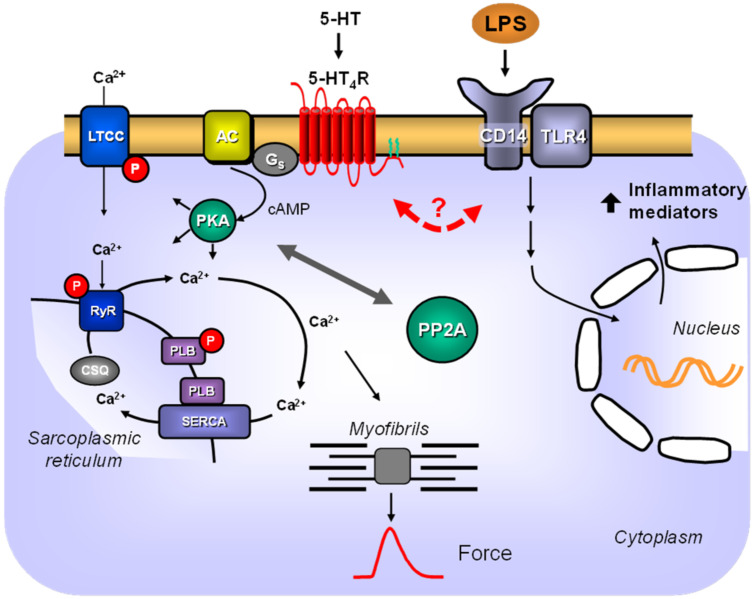
Scheme. 5-HT signaling via 5-HT_4_-receptors and LPS signaling in TG cardiac myocytes. Stimulation of cardiac 5-HT_4_-receptors in the sarcolemma of transgenic mice leads to stimulation of adenylate cyclase (AC) via stimulatory G-proteins (Gs). AC increases cAMP levels in the cytosol, where it can either directly activate HCN channels and thereby increase the beating rate in sinoatrial cells or can activate the cAMP-dependent protein kinase (PKA). PKA can increase Ca^2+^-cycling by phosphorylation of phospholamban (PLB) on serine 16 or of the L-type Ca^2+^ channel (LTCC) or of the ryanodine receptor (RyR). Ca^2+^ is released via the ryanodine receptor, increasing Ca^2+^ levels near the myofibrils, which increases the force of contraction at the beginning of systole. Relaxation is initiated by sarcoplasmic Ca^2+^ ATPase (SERCA), which pumps Ca^2+^ into the sarcoplasmic reticulum at the beginning of diastole. Phosphorylation of these proteins is reduced in part by the catalytic subunit of protein phosphatase 2A (PP2A) and, conversely, the action of PP2A is reduced at least in part by activation of the 5-HT_4_ receptor. Lipopolysaccharide (LPS) can bind to a complex of TLR4 and CD14. This leads via intracellular signaling pathways to increased gene transcription in the nucleus. Here, an interaction between 5-HT_4_ receptor signaling and LPS signaling appears questionable.

Another study performed reversible mechanical occlusion and reperfusion of the LAD in living mice and found increased serum levels of 5-HT in WT mice, but not in TPH1-KO mice; they also found less cardiac damage in the TPH1-KO mice, which indicated that released 5-HT had a detrimental effect in these mice [49]. It is not obvious how our data correlate with the study, as it can be assumed that TPH1-KO mice did not express functional 5-HT_4_ receptors in the heart. Regarding cardiac 5-HT effects, TPH1-KO mice should behave like the WT mice in our study, and we only detected 5-HT_4_ receptors in the sarcolemma of 5-HT_4_-TG mice [20]. Nevertheless, the influence of cardiac 5-HT_4_ receptors during sepsis may only be marginal, at least limited to the scenario we tested (Figure 12). On the other hand, we detected reduced 5-HT_4_ mRNA in transgenic hearts after LPS treatment, and if this suppression was transmitted to the protein and, therefore, functional level, it would not be a surprise that 5-HT_4_-TG mice behave like WT mice after LPS. Unfortunately, when the study was initiated, we were not aware of the possibility that LPS may regulate the transgene and afterward, the protein expression of the 5-HT_4_ receptor cannot be examined because of the missing detection techniques (lack of suitable antibodies for Western blotting). This limitation should be kept in mind for further studies that should include longer LPS treatment and 5-HT stimulation of LPS-treated mice.

### 4.2. Ischemia and Hypoxia

5-HT can be released from ischemic hearts of many species, including rats [50], rabbits [51] and humans with angina pectoris [49,52,53,54]. Our studies produced different results depending on the region of the heart used and the method applied to lower the partial oxygen pressure in the heart. For example, atrial preparations seemed to benefit from 5-HT_4_ receptor overexpression under basal conditions, while 5-HT_4_ receptor overexpression had a detrimental effect on whole perfused hearts. Possibly, these data reflect either a small constitutive activity of the overexpressed 5-HT_4_ receptor or an endogenous serotonin production and release [8], leading to a higher energy and oxygen consumption in transgenic cells compared to WT. It is unclear whether acute ischemia for 20 min would be adequate stress to alter mRNA levels of regulatory genes. However, there is precedence for this in the literature. One study showed that after 10 min occlusion of the LAD artery in anesthetized dogs, the mRNA levels of HSP70 and c-jun increased [55]. Here, it may be regarded as surprising that serine 16 phosphorylation is selectively attenuated after 5-HT infusion following ischemia in the isolated perfused hearts of 5-HT_4_-TG mice. However, this result was also found in a previous study that reported that after a 10 min LAD artery occlusion in anesthetized dogs, the cAMP content and, subsequently, the phosphorylation state of phospholamban at serine 16 was decreased [56]. Thus, these rapid biochemical regulations are consistent in animal studies in larger experimental animals like pigs. In addition, studies have found increased ANP levels in mice with acute regional cardiac ischemia [57]. The overexpression of receptors that stimulate cAMP generation, such as H_2_ receptors [58] or A_2A_-adenosine receptors [35], can protect isolated left atria against acute hypoxia in the same experimental setup. Other studies have reported that cardiac ischemia in mice hearts causes increased IL-6 [59,60]. Diastolic contracture in hypoxia is well known in human atria [61] and mouse hearts [62].

In human end-stage heart failure, the PIE of 5-HT was reduced in human atrial preparations [63]; in addition, the stimulatory effects of 5-HT on adenylyl cyclase [63] and on the current through L-type Ca^2+^ channels were decreased [64]. Moreover, in end-stage heart failure, the density of 5-HT_4_ receptors in the human ventricle was elevated, which may be a compensatory mechanism to reduce β-adrenoceptors in heart failure [11,65]. In a rat model of cardiac hypertrophy, heart failure and cardiac death, the density of 5-HT_4_ receptors was elevated, and the PIE of 5-HT_4_ on the ventricle was unmasked; healthy rats only showed a PIE via 5-HT_2_ receptors [66,67]. In addition, the cardiac cAMP was elevated, and the time parameters of contraction were shortened in the rats [67]. Based on these findings, researchers speculated that 5-HT_4_ receptors might be detrimental in human heart failure because they increase cAMP levels, which can worsen cardiac function in the long run by leading to cardiac hypertrophy. Therefore, they studied a blockade of 5-HT_4_ receptors and noted that the EF in patients with systolic heart failure improved [68].

### 4.3. PP2A-Induced Cardiac Hypertrophy

This study confirmed previously reported results [32]. The cardiomyocyte-specific overexpression of the catalytic subunit of PP2A (PP2Ac) in PP2A-TG mice led to cardiac hypertrophy, signs of systolic and diastolic heart failure and increased left ventricular wall dimensions. We also confirmed that the overexpression of the 5-HT_4_ receptor in the hearts of TG mice alone did not lead to cardiac hypertrophy and/or impaired cardiac function to β-adrenoceptor stimulation [20]. In the present study, the abnormality in diastolic heart function found in the monotransgenic PP2A-TG mice was somewhat reduced in the DT mice. If these functional changes are associated with any morphological changes of DT hearts has not been examined but seems unlikely. Nevertheless, it must be stated that it is a limitation of the study. Although the differences between PP2A-TG and DT mice were either small or non-significant, we carefully regard this as an example of a putative protective role that the 5-HT_4_ receptors have in the heart. This might mean that the 5-HT_4_ receptor, which increases protein phosphorylation under basal conditions [20], can reverse the dephosphorylation of cardiac regulatory proteins (Figure 12). Typically, this is done by PP2Ac alone [32]. Regrettably, we could not test this hypothesis with the set of samples generated by our study protocol because to measure differentially phosphorylated proteins, isolated perfused heart preparations must be prepared and freeze clamped following an exact protocol. This is a drawback of our study. However, one could speculate that this beneficial effect would be seen in patients with elevated serotonin levels as an autocrine way to sustain cardiac contractility. Elevated 5-HT levels have been reported in patients with heart failure [52]. We speculate that 5-HT might, in part, antagonize reduced protein phosphorylation in the hearts of patients with heart failure [69]. Heart failure can be partially explained by increased phosphatase activity [70]. On the other hand, increased phosphorylation by PKA also can be detrimental. For example, this was summarized in a review about the ryanodine receptor found to be PKA hyperphosphorylated, resulting in a defective calcium release in failing human hearts [71]. This once again underlines the need for the right balance between phosphorylation and dephosphorylation.

However, while this work focused on the imbalance of cardiac phosphorylation by overexpression of PP2A as a hypertrophy model, it should be kept in mind that cardiac hypertrophy and/or dysfunction can be experimentally achieved by various methods, including, for example, ablation of 5-HT_2B_ receptors [72,73]. In these studies, the interdependence of serotonin and β-adrenergic signaling, as well as the role of the vegetative nervous system and the cardiac fibroblasts for cardiac hypertrophy and dysfunction, was demonstrated [72,73]. This means that further studies utilizing, for example, other hypertrophy models combined with 5-HT_4_-TG mice are necessary to deepen the knowledge about the relationship (where this exists) between 5-HT_4_ receptors and cardiac disorders.

### 4.4. Conclusions, Potential Relevance in Clinical Pharmacology and Limitations

In summary, our studies suggested that 5-HT_4_ receptor expression in the mouse heart exhibited only marginal effects under pathophysiological conditions like sepsis (Figure 12). However, under special circumstances like atrial hypoxia and ventricular ischemia, cardiac 5-HT_4_ receptor expression may be at least in part detrimental. This needs to be studied further to determine if patients may benefit from treatment with 5-HT_4_ receptor antagonists.

There are several limitations of the study. For example, potential sex differences were not addressed, and examining the morphology of the hearts from the hypertrophy model was excluded in this study. Partially, the study is preliminary and may be regarded as the beginning of more work concerning the molecular mechanisms involved. More data must be generated concerning 5-HT-mediated signaling and the expression of certain genes under pathological conditions. Moreover, the effects of 5-HT_4_ receptor antagonists must be analyzed in this context. Nevertheless, 5-HT_4_-TG mice represent a suitable model to get deeper insights into the role of 5-HT_4_ receptors for cardiovascular diseases.

## Data Availability

The data presented in this study are available on request from the corresponding author.

## Supplementary Materials

The following are available online at https://www.mdpi.com/article/10.3390/biomedicines9050569/s1, Figure S1: Doppler echocardiography of 5-HT_4_-TG mice before and after LPS treatment, Figure S2: hypoxia in spontaneously beating right atria, Figure S3: mRNA expression after ischemia/reperfusion in isolated perfused hearts, Figures S4–S9: unedited Western blots, Tables S1–S3: Echocardiographic data and heart weights.
