# Supplementary material for: Influence of Serotonin 5-HT4 Receptors on Responses to Cardiac Stressors in Transgenic Mouse Models
Source: Biomedicines. 2021 May 18;9(5):569. doi: 10.3390/biomedicines9050569 (PMC8158346; doi:10.3390/biomedicines9050569)
Supplement: Supplementary file 1 [file biomedicines-09-00569-s001.zip › Supplementary Figures.pdf]

Influence of serotonin 5-HT_4 receptors on responses to cardiac stressors in transgenic mouse models

Ulrich Gergs[1], Timo Gerigk[1], Jonas Wittschier[1], Constanze T. Schmidbaur[1], Clara Röttger[1], Mareen Mahnkopf [1], Hanna Edler[1], Hartmut Wache [1], Joachim Neumann [1*]

[1]Institute for Pharmacology and Toxicology, Medical Faculty, Martin Luther University Halle-Wittenberg, Halle (Saale), Germany

Supplementary Figures

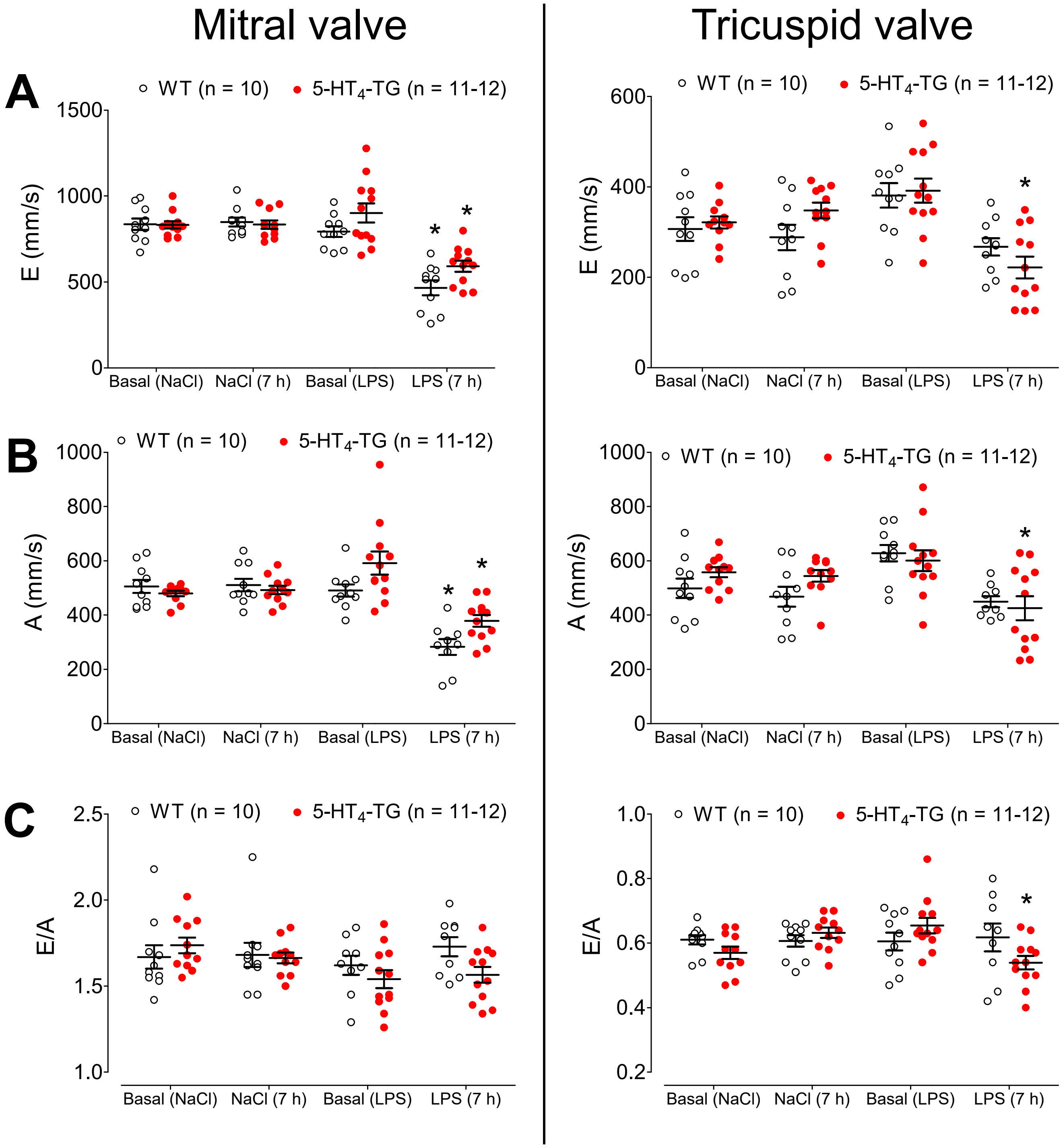

Supplementary Figure S1. Doppler echocardiography of 5-HT_4-TG mice before and after LPS treatment. The flow over the mitral valve (left side) and over the tricuspid valve (right side) was measured before (basal) and 7 h after LPS injection. The injection of a NaCl solution served as control. **(A)** The E wave represents the early filling of the ventricle and **(B)** the A wave represents the atrial contraction. **(C)** The diastolic function expressed as ratio of E and A waves (E/A) was unchanged in all groups. Number in brackets indicates the number of mice studied. WT = wild type mice; *p < 0.05 vs. basal.

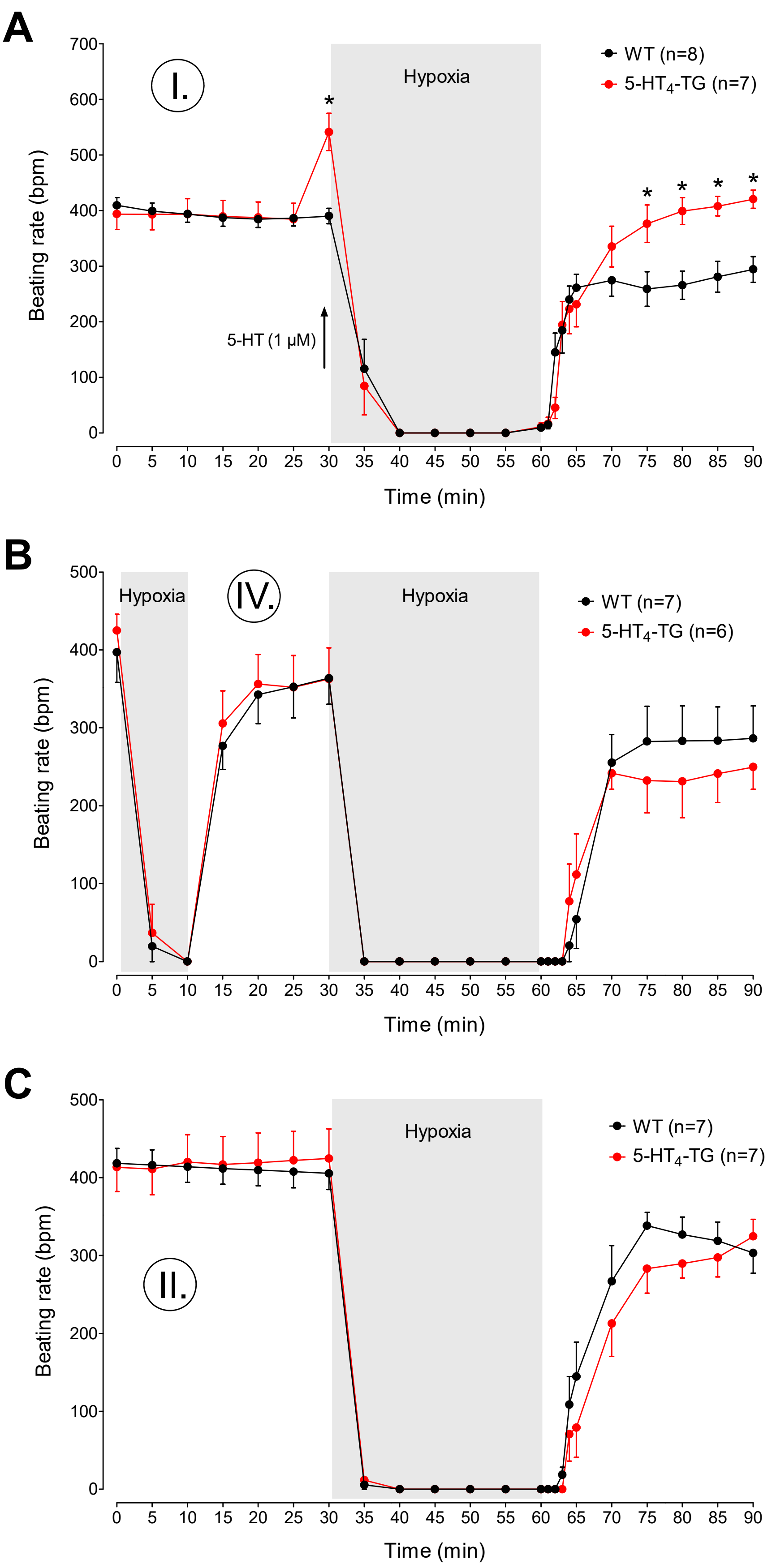

Supplementary Figure S2. Time course of hypoxia in spontaneously beating right atrial preparations. **(A-C)** The beating rate during the time of oxygenation and hypoxia is presented. After the addition of serotonin (5-HT, 1 µM, protocol I) beating rate was increased in 5-HT_4-TG and reached again initial values after hypoxia and reoxygenation **(A)**. Preconditioning (protocol IV) as a short hypoxia for 10 minutes was not beneficial **(B)**. Under the condition of a single hypoxia (protocol II), the beating rate was not different between 5-HT_4-TG and WT right atria **(C)**. WT = wild type mice, 5-HT_4-TG=5-HT_4-transgenic mice. Data shown are means ± SEM. *$p < 0.05$ vs. WT.

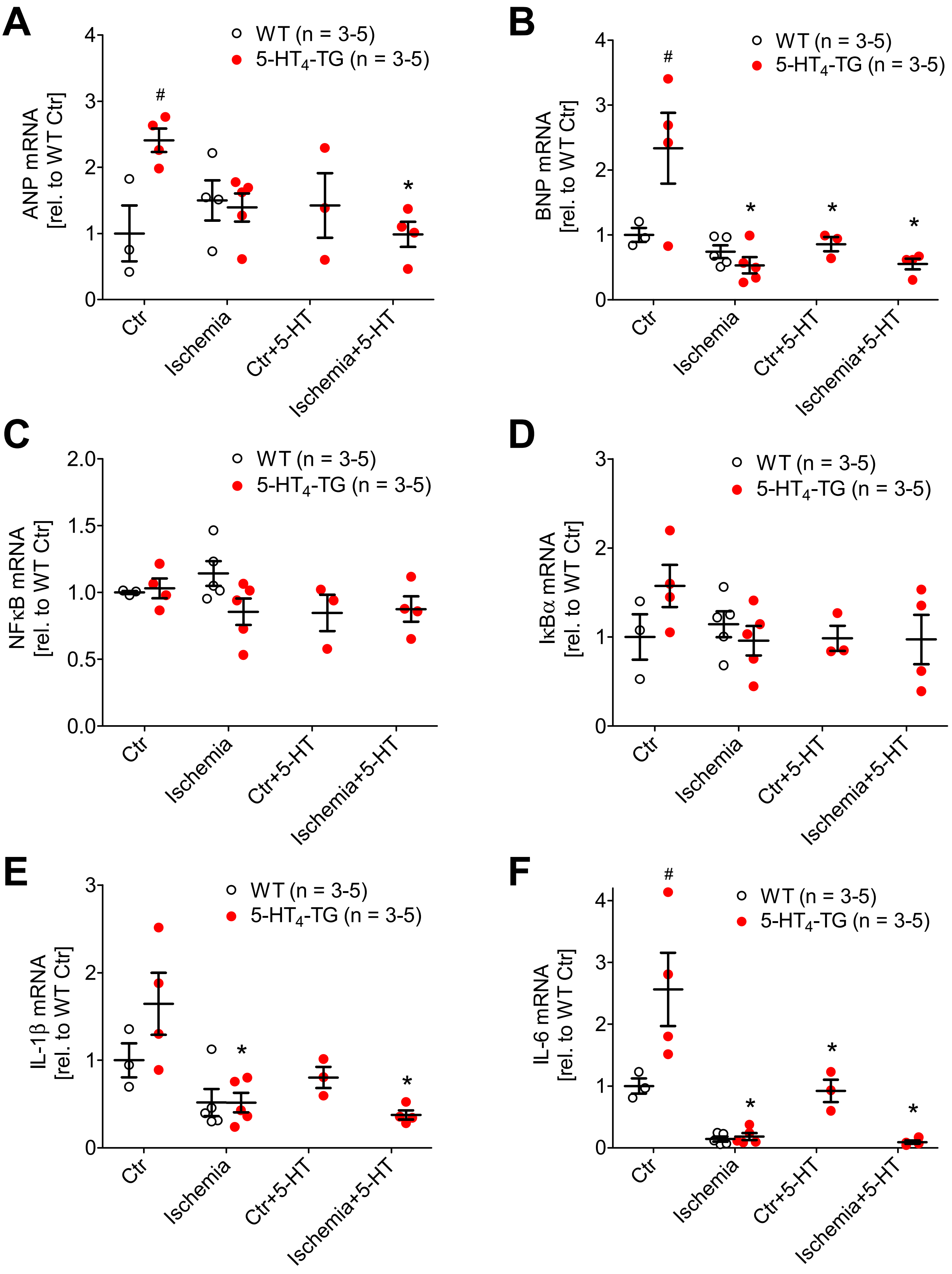

Supplementary Figure S3. mRNA expression after ischemia/reperfusion in isolated perfused hearts. Abundance of mRNAs, measured by quantitative real time PCR (qPCR), after ischemia/reperfusion in isolated perfused hearts of WT und 5-HT_4-TG mice (see Fig. 8A for the protocol). Expression of **(A)** ANP, **(B)** BNP, **(C)** NFκB, **(D)** IκB, **(E)** IL-1β, and **(F)** IL-6 mRNAs in hearts from WT and 5-HT_4-TG mice was normalized to GAPDH expression. Finally, mRNA expression data are presented relative to WT control (Ctr) mice. WT = wild type mice, 5-HT_4-TG=5-HT_4-transgenic mice. Data shown are means ± SEM. *$p < 0.05$ vs. Ctr.; $^{\#}p < 0.05$ vs. WT; numbers in brackets indicate numbers of preparations.

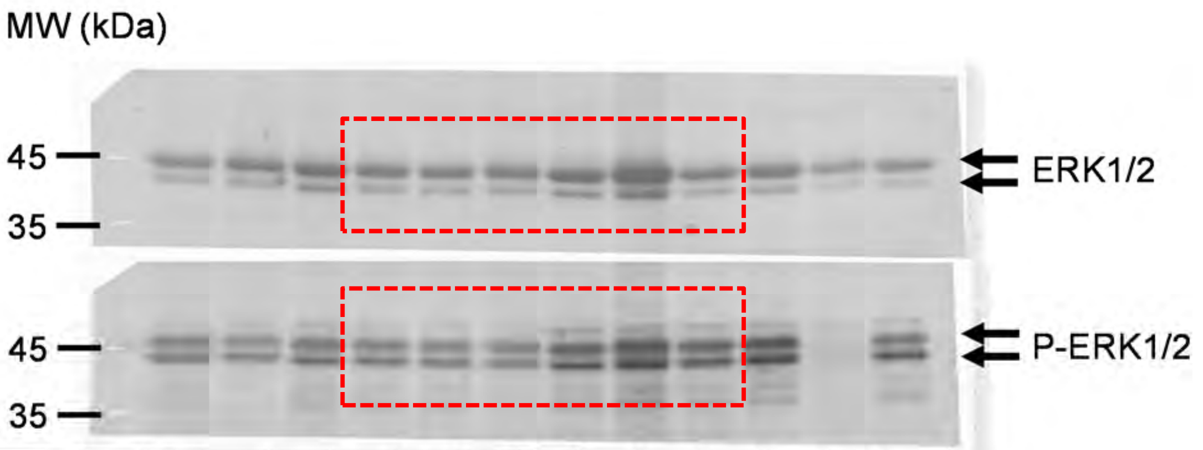

WT	(M)	+		+		+		+				+	+	
5-HT$_4$-TG	(M)		+		+		+		+	+	+			+
Ischemia	(M)			+	+			+	+	+		+		+
5-HT	(M)									+	+			

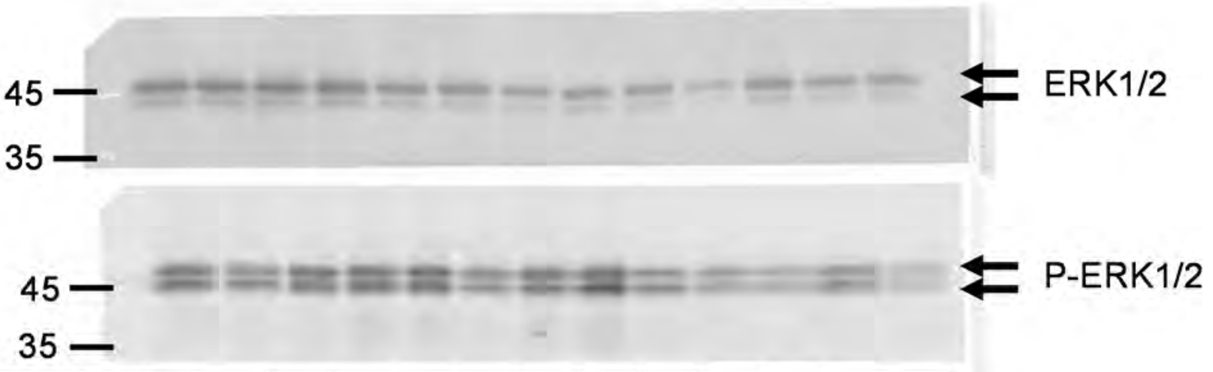

WT	(M)	+		+			+			+		+		+
5-HT$_4$-TG	(M)		+		+	+		+	+		+		+	
Ischemia	(M)			+	+	+			+	+	+			
5-HT	(M)							+	+					

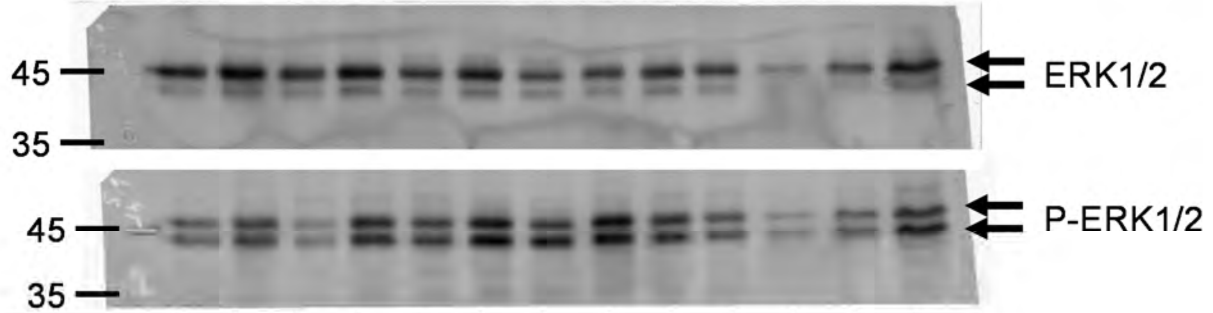

WT	(M)	+		+		+				+	+			
5-HT$_4$-TG	(M)		+		+		+	+	+			+	+	+
Ischemia	(M)	+			+	+	+		+		+			+
5-HT	(M)							+	+					+

Supplementary Figure S4 (unedited Western blots): Original Western blots of homogenates from isolated perfused hearts with or without ischemia of wild type (WT) and 5-HT$_4$ receptor transgenic (5-HT$_4$-TG) mice. M, molecular weight marker; 5-HT, serotonin (1 μM); ERK1/2, extracellular regulated kinase 1/2; P-ERK1/2, phosphorylated ERK1/2. Not all bands were suitable for quantification (border lanes or duplicates). The red rectangles mark the part of the blot shown in Figure 7.

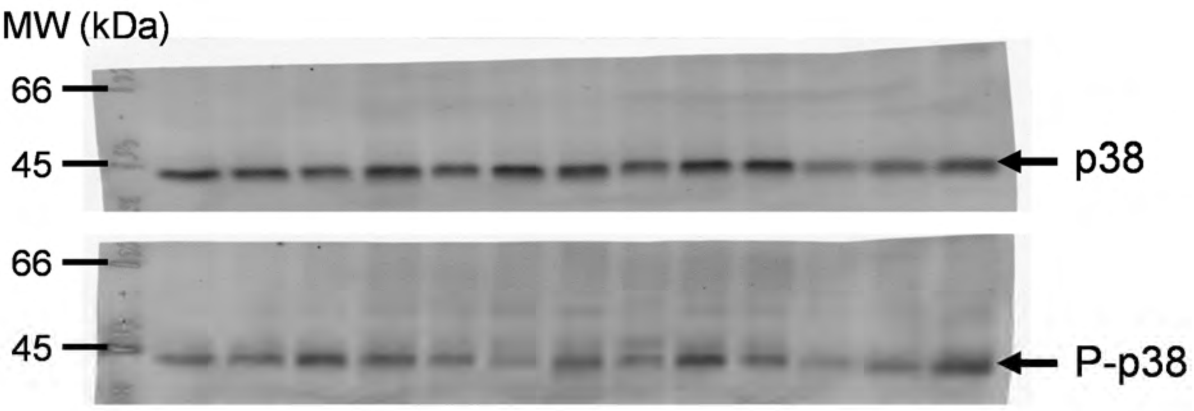

WT	(M)	+		+		+		+				+	+	
5-HT$_4$-TG	(M)		+		+		+		+	+	+			+
Ischemia	(M)			+	+			+		+		+		+
5-HT	(M)									+	+			+

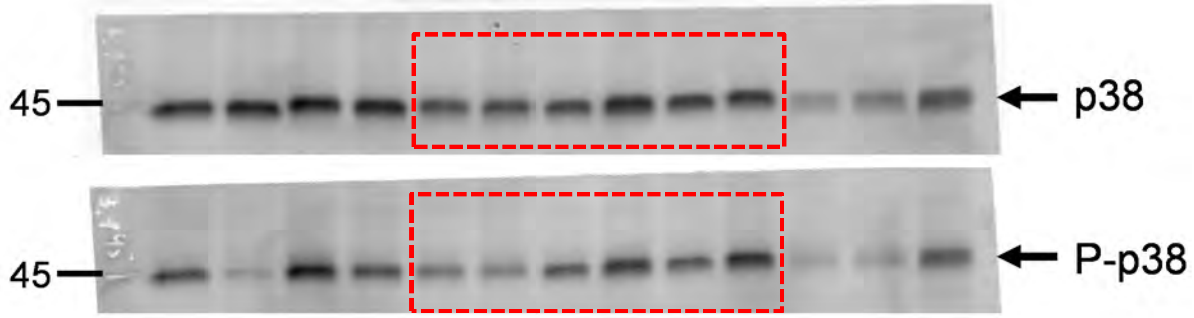

WT	(M)	+				+		+				+		+
5-HT$_4$-TG	(M)		+	+	+		+		+	+	+		+	
Ischemia	(M)		+	+	+			+	+	+			+	
5-HT	(M)									+	+			

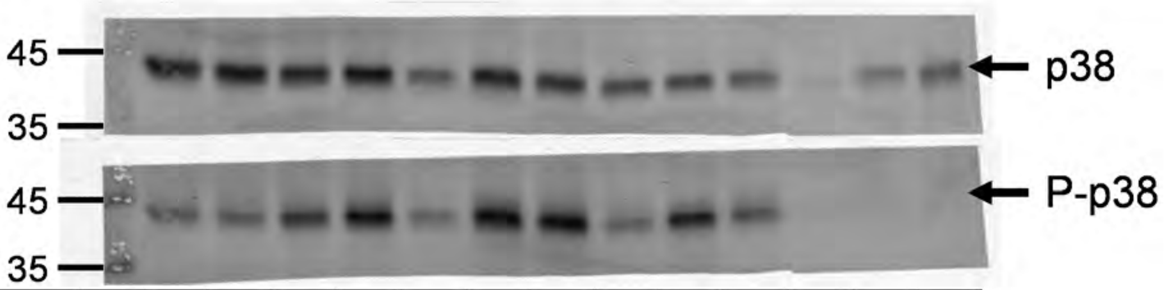

WT	(M)	+		+		+				+	+			
5-HT$_4$-TG	(M)		+		+		+	+	+			+	+	+
Ischemia	(M)	+		+	+			+	+		+			
5-HT	(M)						+	+						

Supplementary Figure S5 (unedited Western blots): Original Western blots of homogenates from isolated perfused hearts with or without ischemia of wild type (WT) and 5-HT$_4$ receptor transgenic (5-HT$_4$-TG) mice. M, molecular weight marker; 5-HT, serotonin (1 μM); p38, mitogen activated protein kinase p38; P-p38, phosphorylated p38. Not all bands were suitable for quantification (border lanes or duplicates). The red rectangles mark the part of the blot shown in Figure 7.

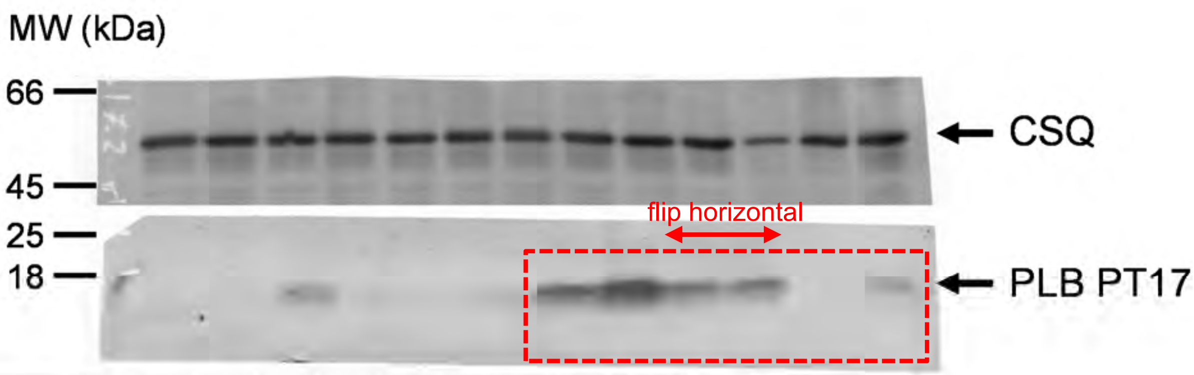

WT	(M)	+				+		+				+		+
5-HT$_4$-TG	(M)		+	+	+		+		+	+	+		+	
Ischemia	(M)		+		+			+		+	+	+		
5-HT	(M)								+	+				

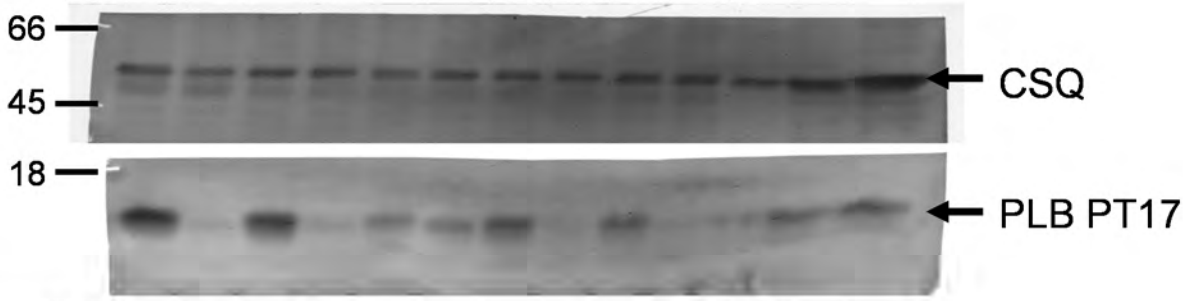

WT	(M)		+		+		+		+		+	+		
5-HT$_4$-TG	(M)	+		+		+		+		+			+	+
Ischemia	(M)			+	+	+	+		+	+	+			
5-HT	(M)			+				+		+				

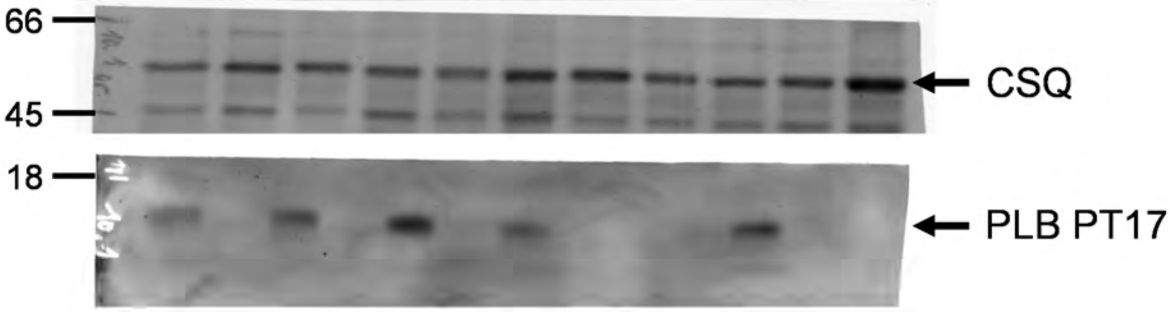

WT	(M)		+		+		+		+	+			+	+
5-HT$_4$-TG	(M)	+		+		+		+			+	+		
Ischemia	(M)			+	+	+					+			
5-HT	(M)					+		+						

Supplementary Figure S6 (unedited Western blots): Original Western blots of homogenates from isolated perfused hearts with or without ischemia of wild type (WT) and 5-HT$_4$ receptor transgenic (5-HT$_4$-TG) mice. M, molecular weight marker; 5-HT, serotonin (1 µM); CSQ, cardiac calsequestrin; PLB PT17, phospholamban phosphorylated at threonine 17. Not all bands were suitable for quantification (border lanes or duplicates). The red rectangle mark the part of the blot shown in Figure 7 after flipping horizontally.

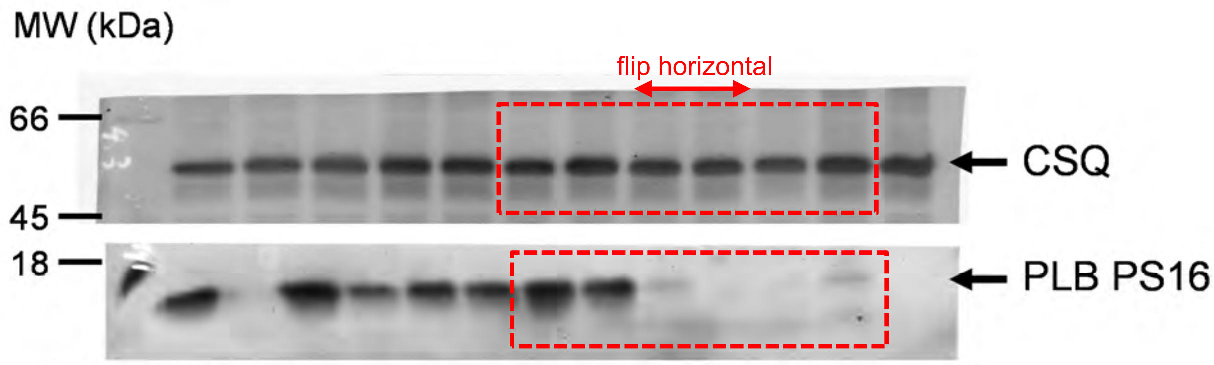

WT	(M)		+		+						+		+	+
5-HT$_4$-TG	(M)	+		+		+	+	+	+	+		+		
Ischemia	(M)		+	+	+				+	+	+			
5-HT	(M)			+				+	+					

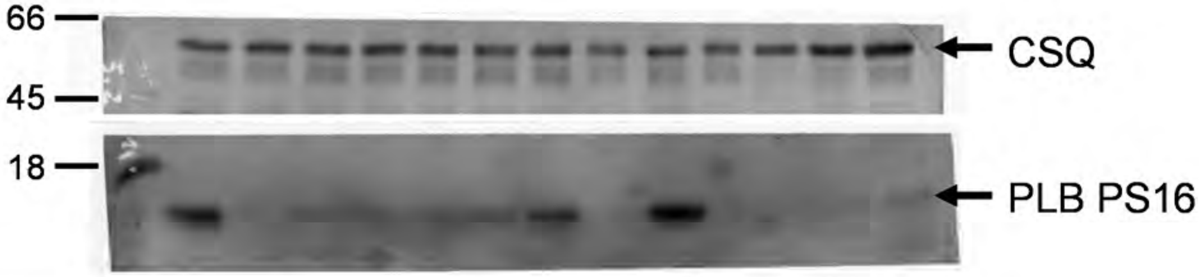

WT	(M)		+		+		+		+		+	+		
5-HT$_4$-TG	(M)	+		+		+		+		+			+	+
Ischemia	(M)			+	+	+	+		+	+	+			
5-HT	(M)			+				+		+				

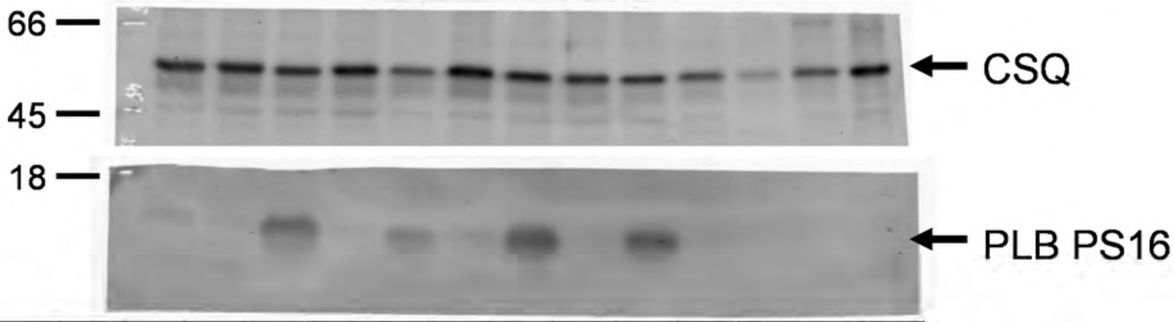

WT	(M)	+	+		+		+		+		+	+	+	+
5-HT$_4$-TG	(M)			+		+		+		+				
Ischemia	(M)	+		+	+					+	+		+	
5-HT	(M)							+		+				

Supplementary Figure S7 (unedited Western blots): Original Western blots of homogenates from isolated perfused hearts with or without ischemia of wild type (WT) and 5-HT$_4$ receptor transgenic (5-HT$_4$-TG) mice. M, molecular weight marker; 5-HT, serotonin (1 µM); CSQ, cardiac calsequestrin; PLB PS16, phospholamban phosphorylated at serine 16. Not all bands were suitable for quantification (border lanes or duplicates). The red rectangles mark the part of the blot shown in Figure 7 after flipping horizontally.

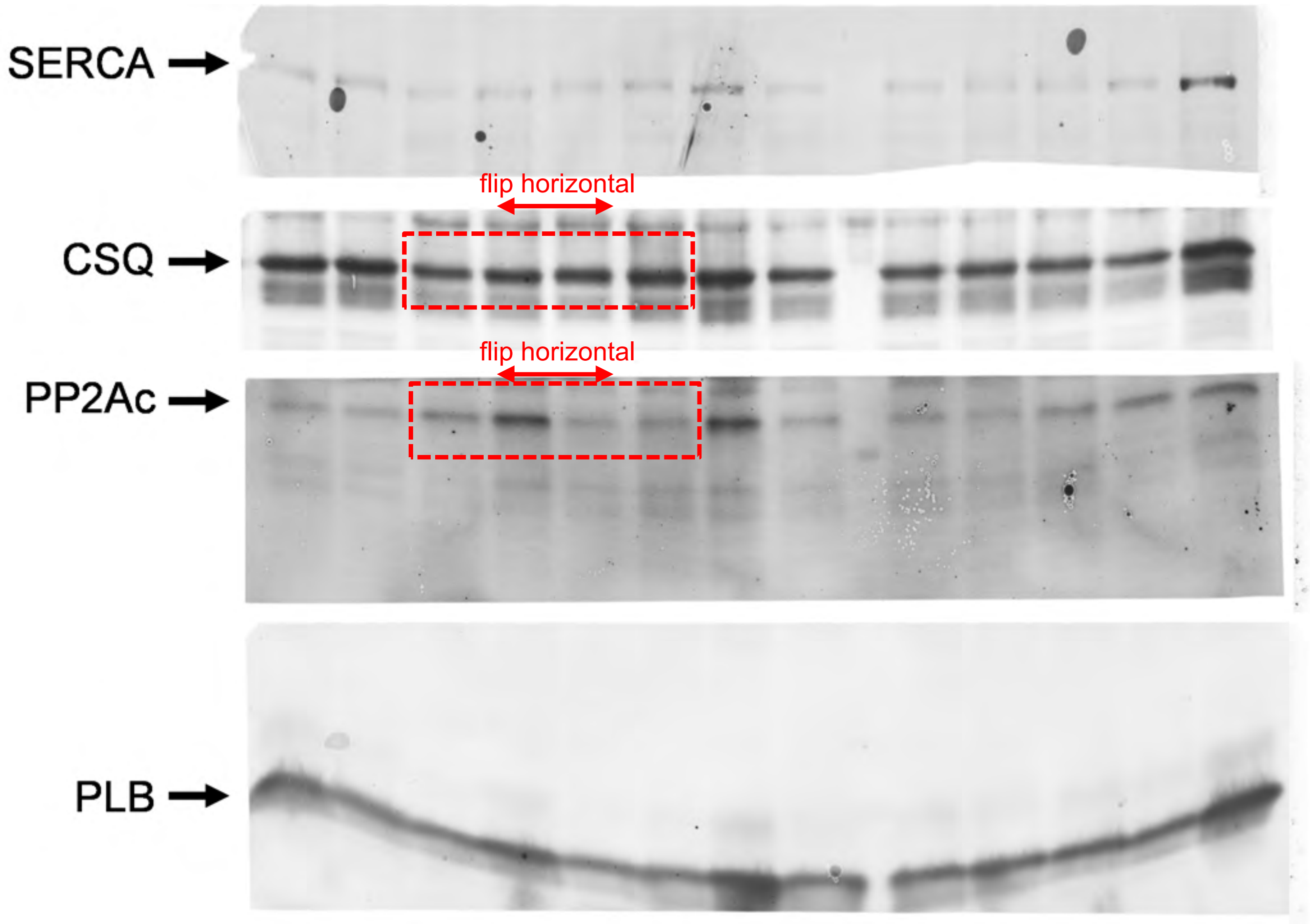

WT	+					+			(M)		+			
5-HT$_4$-TG		+			+				(M)	+				
PP2A-TG				+			+		(M)				+	+
DT			+					+	(M)			+		

Supplementary Figure S8 (unedited Western blots): Original Western blots (Gel 1) of cardiac homogenates from wild type (WT), 5-HT$_4$ receptor transgenic (5-HT$_4$-TG), PP2A transgenic (PP2A-TG), and double transgenic (DT) mice. M, molecular weight marker; CSQ, calsequestrin; SERCA, sarcoplasmic reticulum Ca^{2+} ATPase; PLB, phospholamban; PP2Ac, catalytic subunit of protein phosphatase 2A. The red rectangles mark the parts of the blot shown in the Figure 9 after flipping horizontally.

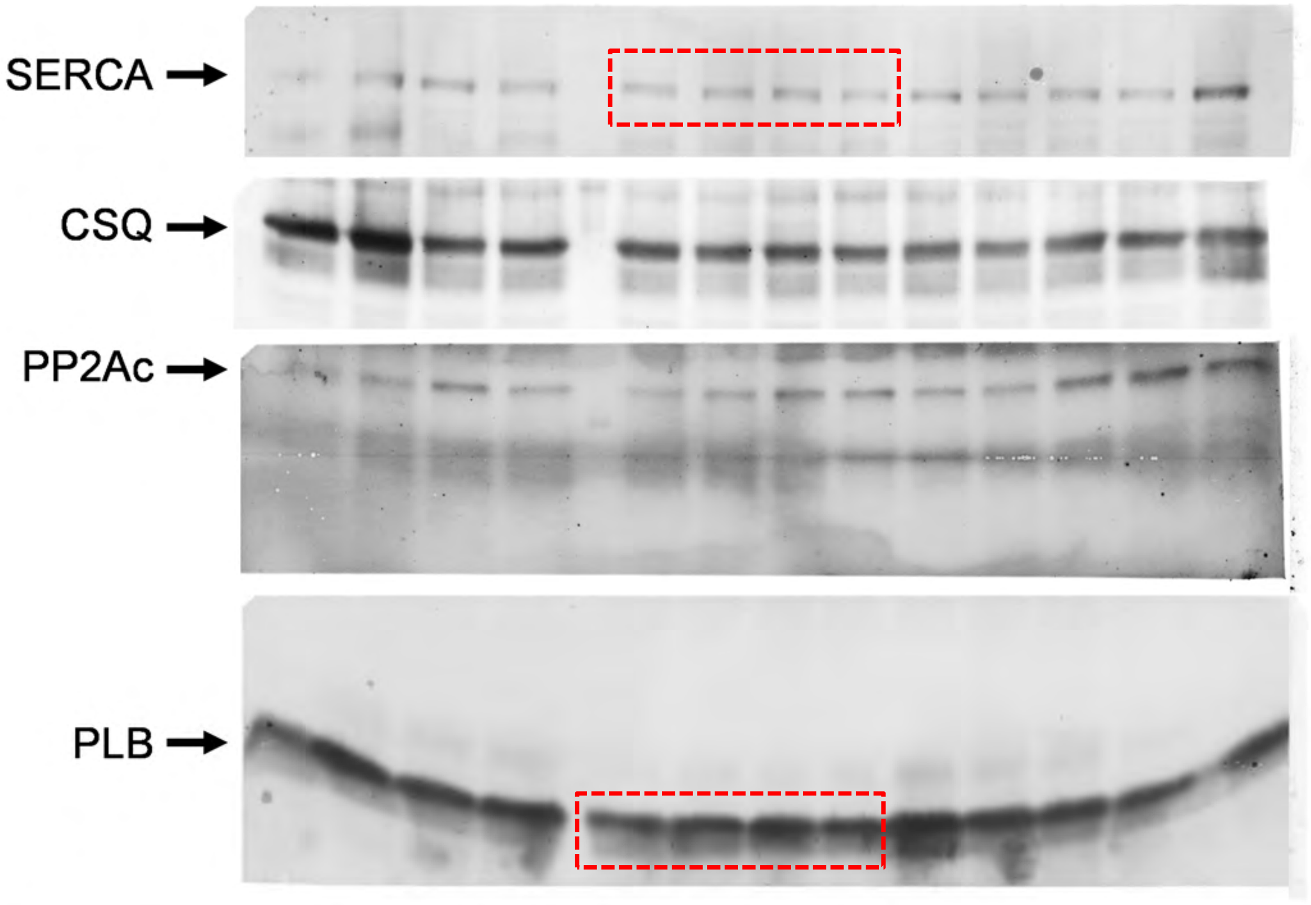

WT	+				(M)	+				+				
5-HT$_4$-TG		+			(M)		+				+			
PP2A-TG			+		(M)			+				+		
DT				+	(M)				+				+	+

Supplementary Figure S9 (unedited Western blots): Original Western blots (Gel 2) of cardiac homogenates from wild type (WT), 5-HT$_4$ receptor transgenic (5-HT$_4$-TG), PP2A transgenic (PP2A-TG), and double transgenic (DT) mice. M, molecular weight marker; CSQ, calsequestrin; SERCA, sarcoplasmic reticulum Ca^{2+} ATPase; PLB, phospholamban; PP2Ac, catalytic subunit of protein phosphatase 2A. The red rectangles mark the parts of the blot shown in the Figure 9.
