# Supplementary material for: Influence of Serotonin 5-HT4 Receptors on Responses to Cardiac Stressors in Transgenic Mouse Models
Source: Biomedicines. 2021 May 18;9(5):569. doi: 10.3390/biomedicines9050569 (PMC8158346; doi:10.3390/biomedicines9050569)
Supplement: Supplementary file 1 [file biomedicines-09-00569-s001.zip › Supplementary Tables.pdf]

Supplementary Table 1. Echocardiography of wild type (WT) and 5-HT$_4$-transgenic (5-HT$_4$-TG) mice before (basal) and seven hours after lipopolysaccharide (LPS) treatment (30 µg/g body weight). NaCl injection served as control. Data shown are means ± SEM of n mice.

	**WT**				**5-HT$_4$-TG**			
	**NaCl**		**LPS**		**NaCl**		**LPS**	
**Parameters**	**basal**	**7 h**	**basal**	**7 h**	**basal**	**7 h**	**basal**	**7 h**
**n**	10	10	10	10	11	11	12	12
**EF (%)**	70.1 ± 0.8	68.4 ± 0.9	70.8 ± 0.9	58.8 ± 6.5*	73.0 ± 0.8	72.5 ± 0.8	72.9 ± 1.7	61.4 ± 4.6*
**HR (bpm)**	461 ± 9.5	442 ± 11.8	487 ± 6.4	588 ± 16.7*	489 ± 12.1	480 ± 9.8	460 ± 10.5	581 ± 22.7*
**Pulsed wave Doppler**								
**Mitral valve**								
**E velocity (mm/s)**	836 ± 31.0	848 ± 24.9	793 ± 28.9	466 ± 41.5*	833 ± 20.9	835 ± 23.1	902 ± 53.8	592 ± 30.7*
**A velocity (mm/s)**	506 ± 23.1	511 ± 21.8	491 ± 21.3	283 ± 27.9*	480 ± 9.3	493 ± 14.4	591 ± 40.3	379 ± 20.4*
**E/A**	1.67 ± 0.06	1.68 ± 0.07	1.63 ± 0.05	1.73 ± 0.05	1.74 ± 0.04	1.66 ± 0.03	1.54 ± 0.05	1.57 ± 0.04
**Tricuspid valve**								
**E velocity (mm/s)**	307 ± 25.8	288 ± 28.2	381 ± 26.9	267 ± 19.1	321 ± 13.3	348 ± 17.3	392 ± 26.3	222 ± 24.2*
**A velocity (mm/s)**	499 ± 35.6	468 ± 36.5	628 ± 30.4	450 ± 20.2	558 ± 18.7	544 ± 21.3	601 ± 38.1	426 ± 44.2*
**E/A**	0.61 ± 0.01	0.61 ± 0.02	0.61 ± 0.03	0.62 ± 0.04	0.57 ± 0.02	0.63 ± 0.02	0.65 ± 0.02	0.54 ± 0.02*
**Peak velocity**								
**Ascending aorta (mm/s)**	1054 ± 52.7	1079 ± 44.4	1158 ± 67.1	783 ± 48.8*	1128 ± 57.5	1143 ± 56.1	1208 ± 67.9	799 ± 49.1*
**Descending aorta (mm/s)**	-975 ± 49.1	-996 ± 43.6	-970 ± 36.1	-589 ± 31.8*	-995 ± 31.8	-989 ± 30.7	-966 ± 76.4	-662 ± 46.8*
**Pulmonary artery (mm/s)**	-656 ± 45.3	-612 ± 60.6	-671 ± 27.2	-453 ± 32.5*	-754 ± 18.0	-762 ± 15.6	-782 ± 40.9	-453 ± 32.5*
**Pulmonary vein**								
**Diastolic velocity (mm/s)**	593 ± 35.6	591 ± 36.0	662 ± 25.6	413 ± 39.4*	631 ± 27.0	633 ± 20.0	683 ± 37.9	491 ± 31.1*
**Systolic velocity (mm/s)**	230 ± 13.8	224 ± 16.0	224 ± 20.3	129 ± 15.4*	215 ± 11.5	210 ± 6.6	250 ± 24.0	144 ± 12.4*
**Superior vena cava**								
**Diastolic velocity (mm/s)**	-298 ± 21.9	-304 ± 24.0	-338 ± 19.7	-184 ± 17.0*	-275 ± 24.5	-286 ± 22.4	-333 ± 32.2	-201 ± 20.2*
**Systolic velocity (mm/s)**	-202 ± 20.6	-207 ± 15.5	-252 ± 11.3	-140 ± 16.5*	-209 ± 26.7	-209 ± 27.4	-229 ± 29.2	-122 ± 11.2*
**Tissue Doppler**								
**E' velocity (mm/s)**	-32.8 ± 2.5	-29.0 ± 2.1	-30.3 ± 1.9	-19.5 ± 1.1*	-31.6 ± 1.7	-30.6 ± 1.4	-32.7 ± 3.0	-22.4 ± 1.7*
**A' velocity (mm/s)**	-22.3 ± 2.0	-19.4 ± 1.5	-21.4 ± 1.7	-13.2 ± 0.9*	-20.0 ± 1.3	-20.3 ± 1.1	-21.0 ± 1.6	-14.0 ± 1.0*
**E'/A'**	1.49 ± 0.03	1.48 ± 0.03	1.45 ± 0.07	1.47 ± 0.04	1.60 ± 0.03	1.55 ± 0.04	1.56 ± 0.05	1.72 ± 0.12

EF, ejection fraction; HR, heart rate; *p < 0.05 vs. basal

Supplementary Table 2. Characteristics of experimental mice. Body weight and heart weight (absolute and relative) of wild type (WT), 5-HT_4-TG, PP2A-TG and double transgenic (DT) mice are given. The relative weights were calculated as organ weight/body weight. TG, transgenic mice. Data shown are means ± SEM.

Parameters	WT	5-HT_4-TG	PP2A-TG	DT
**n**	6	7	7	8
**Body weight (g)**	40.7 ± 3.3	40.8 ± 2.8	42.8 ± 2.0	41.3 ± 1.6
**Heart weight (mg)**	177.38 ± 16.74	207.83 ± 19.10	259.71 ± 17.19$^{\#}$	229.01 ± 18.53
**LA weight (mg)**	3.78 ± 0.42	6.61 ± 1.10	8.09 ± 1.27$^{\#}$	5.89 ± 0.40
**RA weight (mg)**	4.93 ± 0.53	6.73 ± 1.15	8.63 ± 1.69	7.08 ± 0.68
**Ventricle weight (mg)**	163.33 ± 15.16	188.61 ± 18.30	234.03 ± 15.51$^{\#}$	206.66 ± 16.18
**Relative heart weight**	4.36 ± 0.21	5.06 ± 0.20	6.13 ± 0.51$^{\#}$	5.51 ± 0.32
**Relative LA weight**	0.10 ± 0.01	0.16 ± 0.02	0.19 ± 0.02$^{\#}$	0.14 ± 0.01
**Relative RA weight**	0.12 ± 0.01	0.16 ± 0.02	0.20 ± 0.03	0.17 ± 0.01
**Relative ventricle weight**	4.02 ± 0.16	4.58 ± 0.20	5.54 ± 0.49$^{\#}$	4.98 ± 0.27

LA, left atrium; RA, right atrium; $^{\#}p < 0.05$ vs. WT

Supplementary Table 3. Echocardiography of wild type (WT), 5-HT$_4$-transgenic (5-HT$_4$-TG), PP2A-transgenic (PP2A-TG) and double transgenic (DT) mice. Data shown are means ± SEM of n mice.

	**WT**			**5-HT$_4$-TG**			**PP2A-TG**			**DT**		
**Parameters**	**basal**	**5-HT**	**Iso**	**basal**	**5-HT**	**Iso**	**basal**	**5-HT**	**Iso**	**basal**	**5-HT**	**Iso**
**n**	18	7	7	18	8	8	7	7	7	10	10	10
**EF (%)**	71.1 ± 1.2	78.2 ± 3.5	98.7 ± 0.8*	65.9 ± 1.5	97.0 ± 2.2*$^{\#}$	97.8 ± 1.1*	39.9 ± 3.4$^{\S\#}$	45.5 ± 3.6$^{\S\#}$	78.4 ± 3.1*$^{\S\#}$	39.8 ± 2.1$^{\S\#}$	59.9 ± 5.2*$^{\S\#+}$	68.1 ± 5.5*$^{\S\#}$
**HR (bpm)**	494 ± 17	509 ± 47	605 ± 31*	455 ± 7	538 ± 17*	546 ± 17*	467 ± 11	466 ± 14	528 ± 20$^{\#}$	473 ± 13	521 ± 17*	537 ± 19
**Pulsed wave Doppler**												
**Mitral valve**												
**E velocity (mm/s)**	808 ± 24.7	n.d.	n.d.	846 ± 31.5	n.d.	n.d.	757 ± 55.6	n.d.	n.d.	665 ± 27.2$^{\S\#}$	n.d.	n.d.
**A velocity (mm/s)**	483 ± 11.4	n.d.	n.d.	491 ± 25.8	n.d.	n.d.	333 ± 62.8$^{\S\#}$	n.d.	n.d.	313 ± 41.2$^{\S\#}$	n.d.	n.d.
**E/A**	1.68 ± 0.05	n.d.	n.d.	1.76 ± 0.06	n.d.	n.d.	1.96 ± 0.08$^{\#}$	n.d.	n.d.	1.90 ± 0.09$^{\#}$	n.d.	n.d.
**Tricuspid valve**												
**E velocity (mm/s)**	304 ± 16.8	n.d.	n.d.	261 ± 22.2	n.d.	n.d.	197 ± 23.5$^{\#}$	n.d.	n.d.	233 ± 25.9$^{\#}$	n.d.	n.d.
**A velocity (mm/s)**	530 ± 25.1	n.d.	n.d.	435 ± 33.0$^{\#}$	n.d.	n.d.	334 ± 38.1$^{\#}$	n.d.	n.d.	369 ± 39.5$^{\#}$	n.d.	n.d.
**E/A**	0.57 ± 0.02	n.d.	n.d.	0.60 ± 0.02	n.d.	n.d.	0.57 ± 0.03	n.d.	n.d.	0.63 ± 0.04	n.d.	n.d.
**Peak velocity**												
**Ascending aorta (mm/s)**	1032 ± 60	909 ± 112	1264 ± 115	1098 ± 44	1382 ± 139$^{\#}$	1459 ± 129*	837 ± 74§	824 ± 73§	1106 ± 132§	862 ± 55§	1118 ± 72^{+}	1248 ± 99*
**Tissue Doppler**												
**E' velocity (mm/s)**	-27.7 ± 1.9	n.d.	n.d.	-30.2 ± 2.7	n.d.	n.d.	-32.8 ± 4.8	n.d.	n.d.	-30.9 ± 4.6	n.d.	n.d.
**A' velocity (mm/s)**	-19.1 ± 0.9	n.d.	n.d.	-20.6 ± 1.8	n.d.	n.d.	-16.7 ± 4.1	n.d.	n.d.	-18.2 ± 2.9	n.d.	n.d.
**E'/A'**	1.43 ± 0.07	n.d.	n.d.	1.48 ± 0.07	n.d.	n.d.	1.93 ± 0.19$^{\S\#}$	n.d.	n.d.	1.70 ± 0.16	n.d.	n.d.

EF, ejection fraction; HR, heart rate; n.d., not determined; *$p < 0.05$ vs. basal; $^{\#}p < 0.05$ vs. WT; $^{\S}p < 0.05$ vs. 5-HT$_4$-TG; $^{+}p < 0.05$ vs. PP2A-TG
